# Cavity-excited Huygens' metasurface antennas for near-unity aperture illumination efficiency from arbitrarily large apertures

**DOI:** 10.1038/ncomms10360

**Published:** 2016-01-21

**Authors:** Ariel Epstein, Joseph P. S. Wong, George V. Eleftheriades

**Affiliations:** 1 The Edward S. Rogers Department of Electrical and Computer Engineering, University of Toronto, Toronto, Ontario, Canada M5S 2E4

## Abstract

One of the long-standing problems in antenna engineering is the realization of highly directive beams using low-profile devices. In this paper, we provide a solution to this problem by means of Huygens' metasurfaces (HMSs), based on the equivalence principle. This principle states that a given excitation can be transformed to a desirable aperture field by inducing suitable electric and (equivalent) magnetic surface currents. Building on this concept, we propose and demonstrate cavity-excited HMS antennas, where the single-source-fed cavity is designed to optimize aperture illumination, while the HMS facilitates the current distribution that ensures phase purity of aperture fields. The HMS breaks the coupling between the excitation and radiation spectra typical to standard partially reflecting surfaces, allowing tailoring of the aperture properties to produce a desirable radiation pattern, without incurring edge-taper losses. The proposed low-profile design yields near-unity aperture illumination efficiencies from arbitrarily large apertures, offering new capabilities for microwave, terahertz and optical radiators.

## Abstract (web summary)

The realization of highly-directive beams using low-profile devices has been a long-standing problem. Here, Epstein *et al.* demonstrate a cavity-excited Huygens' metasurface antenna, where the single-source-fed cavity is designed to optimize aperture illumination, while the metasurface facilitates phase purity.

## Graphical abstract

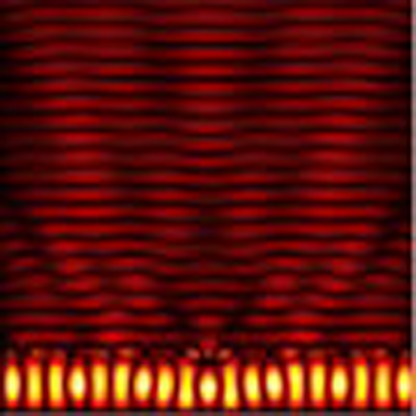

Achieving high directivity with compact radiators has been a major problem in antenna science since early days^1^,^2^,^3^. Still today, many applications, such as automotive radars and satellite communication, strive for simple and efficient low-profile antennas producing the narrowest beams^4^,^5^,^6^,^7^. Increasing the radiating aperture size enhances directivity, but only if the aperture is efficiently excited. To date, uniform illumination of large apertures is achievable with reflectors and lenses; however, these require substantial separation between the source and the aperture, resulting in a large overall antenna size^8^,^9^. High aperture illumination efficiencies can also be achieved using antenna arrays^10^, but the elaborated feed networks increase complexity and cost, and can lead to high losses^11^.

Contrarily, leaky-wave antennas (LWAs) can produce directive beams using a low-profile structure fed by a simple single source^12^. In Fabry–Pérot (FP) LWAs, a localized source is sandwiched between a perfect electric conductor (PEC) and a partially reflecting surface (PRS), forming a longitudinal FP cavity^2^,^13^. By tuning the cavity dimensions and source position, favourable coupling to a single waveguided mode is achieved, forming a leaky wave emanating from the source; typical device thicknesses lie around half of a wavelength. The leaky mode is characterized by a transverse wavenumber whose real-part *k*_t_ corresponds to the waveguide dispersion, and is accompanied by a small imaginary part *α* determined by the PRS. Assuming 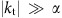, this leads to a conical directive radiation through the PRS towards *θ*_out_≈±arcsin(|*k*_t_|/*k*), 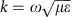 being the free-space wavenumber, with a beamwidth proportional to *α*. Broadside radiation is achieved when |*k*_t_| is small enough such that the splitting condition |*k*_t_|<*α* is satisfied, and the peaks of the conical beam merge^14^. LWAs based on modulated metasurfaces (MoMetAs) are also compact and probe-fed, but utilize a surface wave |*k*_t_|>*k* guided on a PEC-backed dielectric sheet covered with metallic patches^15^,^16^,^17^,^18^. This mode is coupled to radiation via periodic modulation of the patch geometry; its leakage rate *α* is determined by the modulation depth^18^.

Although FP-LWAs and MoMetAs have compact configurations, they suffer from a fundamental efficiency limitation for finite structures: designing a moderate leakage rate *α* yields uniform illumination but results in considerable losses from the edges; on the other hand, for large leakage rates only a portion of the aperture is effectively radiating^19^,^20^,^21^.

To mitigate edge-taper losses, shielded FP-LWA structures have been recently proposed, using PEC side walls which form a lateral cavity^22^,^23^,^24^,^25^,^26^,^27^,^28^. Nevertheless, the tight coupling between the propagation of the leaky mode inside the FP cavity and the angular distribution of the radiated power manifested by *θ*_out_≈arcsin(*k*_t_/*k*) poses serious limitations on the achievable aperture illumination efficiency. This is most prominent for antennas radiating at broadside, in which only low-order lateral modes (satisfying the splitting condition) can be used. Consequently, such antennas are designed to excite exclusively the TE_10_ lateral mode, which inherently limits the aperture illumination efficiency, defined as the relative directivity with respect to the case of uniform illumination, to 81% (ref. 29). In addition, as the dominant spectral components of the cavity fields directly translate to prominent lobes in the radiation pattern, only a single mode should be excited to guarantee high directivity. However, suppression of parasitic cavity modes is a very difficult problem^30^, especially for large apertures.

From the so far discussion it follows that it would be very beneficial if the fields inside the cavity and those formed on the aperture could be optimized independently. This would facilitate good aperture illumination without the necessity to meet excitation-related restricting conditions. But how to achieve such a separation? The equivalence principle suggests that for a given field exciting a surface, desirable (arbitrary) aperture fields can be formed by inducing suitable electric and (equivalent) magnetic surface currents^29^. On the basis of this idea, the concept of Huygens' metasurfaces (HMSs) has been recently proposed, where subwavelength electric and magnetic polarizable particles (meta-atoms) are used to generate these surface currents in response to a known incident field^31^,^32^,^33^,^34^,^35^,^36^,^37^,^38^,^39^. In previous work, we have shown that if the reflected and transmitted fields are properly set, the aperture phase can be tailored by a passive and lossless HMS to produce prescribed directive radiation, for any given excitation source^40^.

In this paper, we harness the equivalence principle to efficiently convert fields excited in a cavity by a localized source to highly directive radiation using a Huygens' metasurface: cavity-excited HMS antenna. The device structure resembles a typical shielded FP-LWA, with an electric line source surrounded by three PEC walls and a HMS replacing the standard PRS (Fig. 1). For a given aperture length *L* and a desirable transmission angle *θ*_out_, we optimize the cavity thickness and source position to predominantly excite a high-order lateral mode, thus guaranteeing good aperture illumination. Once the source configuration is established, we stipulate the aperture fields to follow the power profile of the cavity mode, and impose a linear phase to promote radiation towards *θ*_out_. With the cavity and aperture fields in hand, we invoke the equivalence principle and evaluate the (purely reactive^40^) electric surface impedance and magnetic surface admittance required to support the resultant field discontinuity^31^,^32^,^41^,^42^. As the power profile of the chosen high-order mode creates hot spots of radiating surface currents approximately half a wavelength apart, a uniform virtual phased array is formed on the HMS aperture; such excitation profile is expected to yield very high directivity with no grating lobes regardless of *θ*_out_ (ref. 10). Furthermore, in contrast to LWAs, the antenna directivity does not deteriorate significantly even if other modes are partially excited, as these would merely vary the amplitude of the virtual array elements, without affecting the phase purity. This semianalytical design procedure can be applied to arbitrarily large apertures, yielding near-unity aperture illumination efficiencies. With the PEC side walls, no power is lost via the edges, offering an effective way to overcome the efficiency tradeoff inherent to FP-LWAs and MoMetAs, while preserving the advantages of a single-feed low-profile antenna.

## Results

### Cavity-excited Huygens' metasurface antennas

**Figure 1:**
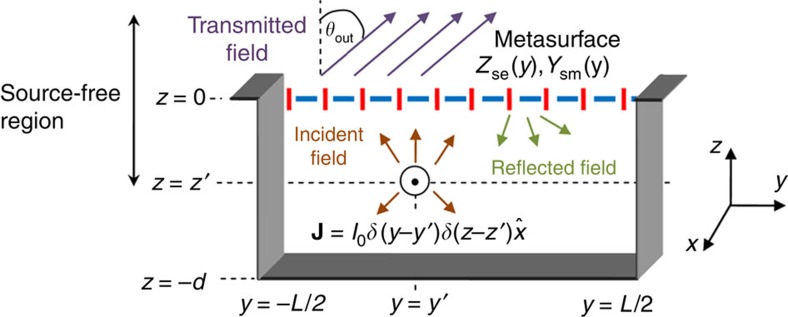
Physical configuration of a cavity-excited Huygens' metasurface antenna. An electric line source is positioned at (y′,z′), surrounded by three perfect-electric-conductor (PEC) walls at forming a lateral cavity. The cavity is covered by a Huygens' metasurface of aperture length L situated at z=0, facilitating directive radiation towards.

To design the HMS-based antenna, we apply the general methodology developed in ref. 40 to the source configuration of Fig. 1; for completeness, we recall briefly its main steps. We consider a two-dimensional (2D) scenario (∂/∂*x*=0) with the HMS at *z*=0 and a given excitation geometry at *z*≤*z*′<0 embedded in a homogeneous medium (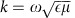, 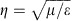). Under these circumstances, the incident, reflected and transmitted fields in the vicinity of the HMS can be expressed via their plane-wave spectrum^43^


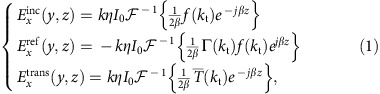


where 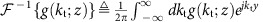 is the inverse spatial Fourier transform of *g*(*k*_t_;*z*) (ref. 44), *f*(*k*_t_) is the source spectrum, Γ(*k*_t_) is the HMS reflection coefficient, and 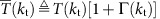 is the transmission spectrum. As before, *k*_t_ denotes the transverse wavenumber and the longitudinal wavenumber is 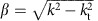. For simplicity, we only consider here transverse electric (TE) fields (*E*_*z*_=*E*_*y*_=*H*_*x*_=0); the nonvanishing magnetic field components *H*_*y*_, *H*_*z*_ can be calculated from *E*_*x*_ via Maxwell's equations.

For a given source spectrum, it is required to determine the reflected and transmitted fields, through the respective degrees of freedom Γ(*k*_t_) and *T*(*k*_t_), that would implement the desirable functionality. Once the tangential fields on the two facets of the HMS are set, the equivalence principle is invoked to evaluate the required electric and magnetic surface currents to induce them^29^. The polarizable particles comprising the HMS are then designed such that the average fields acting on them effectively induce these surface currents^41^,^42^. Analogously, the HMS can be characterized by its electric surface impedance *Z*_se_(*y*) and magnetic surface admittance *Y*_sm_(*y*), relating the field discontinuity and the average excitation via the generalized sheet transition conditions (GSTCs)^31^,^32^,^40^,^41^.

To promote directive radiation towards *θ*_out_ we require that the aperture (transmitted) fields approximately follow the suitable plane-wave-like relation (Supplementary Note 1)


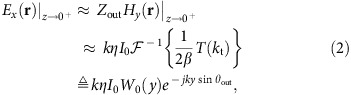


where *W*_0_(*y*) is the aperture window (envelope) function (yet to be determined) and *Z*_out_=1/*Y*_out_=*η*/cos *θ*_out_ is the TE wave impedance of a plane-wave directed towards *θ*_out_.

In previous work^40^, we have shown that if the wave impedance and the real power are continuous across the metasurface, then these aperture fields can be supported by a passive lossless HMS (purely reactive *Z*_se_ and *Y*_sm_). The first condition, local impedance equalization, means that the total (incident+reflected) fields on the bottom facet of the metasurface should exhibit the same wave impedance as the aperture fields, that is, *E*_*x*_(**r**)|_*z*→0^−^_=*Z*_out_*H*_*y*_(**r**)|_*z*→0^−^_; this is achieved by setting the reflection coefficient to a Fresnel-like form


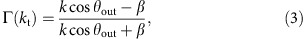


determining the reflected fields everywhere, fixing our first degree of freedom.

To satisfy the second condition, local power conservation, we require that the aperture window function follows the magnitude of the total (incident+reflected) fields at *z*→0^−^, namely,


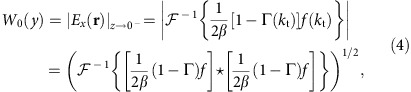


where g*g is the autocorrelation of the spectral-domain function *g*(*k*_t_) (ref. 44); this determines the transmitted fields everywhere, fixing our second degree of freedom.

The absolute value operator in the last equality is of utmost significance: it indicates that the transmission spectrum of the aperture fields follows, up to a square root, the power spectral density of *E*_*x*_(**r**)|_*z*→0^−^_, and not the spectral content of the incident and reflected fields. This is directly related to the balanced (plane-wave-like) contribution of the electric and magnetic fields to the power flow that we stipulated in equation (2), and results in a significantly favourable plane-wave spectrum, as will be discussed in detail in the next section.

Finally, we use these semianalytically predicted fields and the equivalence principle, manifested by the GSTCs, to calculate the required HMS surface impedance, yielding the desirable purely reactive modulation given by^40^,


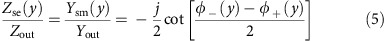


where 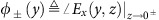 are the phases of the stipulated fields just above and below the metasurface.

Once the general design procedure is established, applying it to the configuration of Fig. 1, which includes an electric line source at (*y* ′, *z* ′) surrounded by PEC walls at *z*=−*d*, *y*=±*L*/2, is straightforward: it is reduced to finding the corresponding source spectrum. The latter is quantized due to the lateral cavity, and includes multiple reflections between the HMS at *z*=0 and the PEC at *z*=−*d*; explicitly^43^,


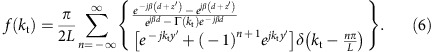


We refer to the sum of the fields corresponding to the *n*, −*n* terms in the summation as the field of the *n*th mode of the lateral cavity, where *n*≥0.

Although this procedure is applicable for any transmission angle, we restrict ourselves from now on to the case of broadside radiation *θ*_out_=0, where the performance of shielded and unshielded FP-LWAs is the most problematic due to the splitting condition^14^ (design of oblique-angle radiators is addressed in the Supplementary Methods). For simplicity, we set the lateral position of the source to be *y* ′=0; with this choice, the even modes vanish, and the odd modes follow a cosine profile in the lateral dimension.

### Optimizing the cavity excitation

One of the key differences between the cavity-excited HMS antenna and FP-LWAs is that by harnessing the equivalence principle we control the individual contributions of the electric and magnetic fields to the flow of power, expressed by the lateral distribution of the *z*-component of the Poynting vector on the aperture. More specifically, the resultant (transmitted) aperture fields corresponding to equation (4) actually follow the square root of the power profile dictated by the cavity mode, and not the profile of the cavity fields. This distinction is very important, as the power profile of a standing wave is always positive, whereas the field profile changes signs along the lateral dimension. Hence, the spectral content of the aperture fields, which determines the far-field radiation pattern, is fundamentally different.

**Figure 2:**
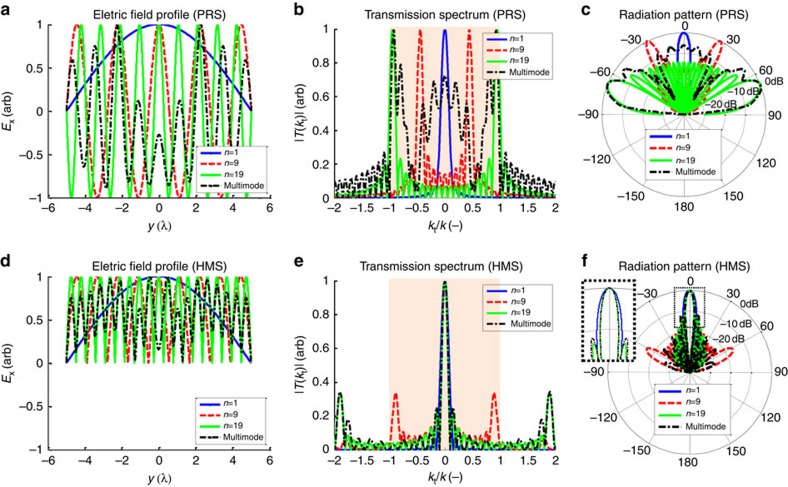
Comparison between aperture profiles and radiation patterns of cavity-excited PRSs and cavity-excited HMSs. Single-mode excitations of the *n*=1 (blue solid line), *n*=9 (red dashed line), and *n*=19 (green solid line) modes of an aperture of length *L*=10*λ* are compared to the multimode excitation corresponding to the HMS antenna presented in Fig. 1 with *L*=10*λ*, *z*′=−*λ*, and *d*=1.61*λ* (black dash-dotted line). (**a**,**d**) Normalized spatial profile of the tangential electric field on the aperture. (**b**,**e**) Normalized spectral content of the aperture field; shaded region correspond to the visible part of the spectrum. (**c**,**f**) Normalized radiation patterns. Inset: close-up of the radiation pattern around *θ*=0.

To illustrate this point, we compare the fields formed on the device aperture for a shielded FP-LWA, where a standard PRS is used, and for a cavity-excited HMS antenna with the same excitation. Figure 2 presents the spatial profile of the tangential electric field, its spatial Fourier transform, and corresponding radiation patterns (calculated following ref. 29), for single-mode excitation of the *n*=1 (blue solid lines), *n*=9 (red dashed lines) and *n*=19 (green solid lines) modes, for an aperture length of *L*=10*λ*. All plots are normalized to their maximum, as the radiation pattern is sensitive to the variation of the fields, and not to their magnitude.

As follows from equations (4) and (6), the spatial profile of the *n*th-mode aperture field is proportional to cos (*nπy*/*L*) for a standard PRS, but for an HMS it is proportional to |cos (*nπy*/*L*)| (Fig. 2a,d). Except for the lowest order mode *n*=1, for which the two functions coincide, the difference in the spatial profile translates into distinctively different features in the spectral content (Fig. 2b,e). For the *n*th mode, the transmission spectrum of the HMS aperture corresponds to the autocorrelation of the PRS aperture spectrum, leading to formation of peaks centred around the second harmonics (*k*_t_=±2*nπ*/*L*) and d.c. (*k*_t_=0). As both the right-propagating and left-propagating components of the standing wave coherently contribute to the d.c. peak, the latter dominates the transmission spectrum, and the radiation patterns corresponding to the HMS aperture exhibit highly directive radiation towards broadside (Fig. 2f). In contrast, the PRS-based devices exhibit conical radiation to angles determined by the dominant spectral components of the aperture fields, that is, towards *θ*=±arcsin[*nλ*/(2*L*)] (Fig. 2c)^12^,^13^.

The transverse wavenumber *k*_t_=*π*/*L* corresponding to the lowest order mode *n*=1 is small enough such that the two symmetric beams merge^14^, which enables the PRS aperture to radiate a single beam at broadside. Indeed, small-aperture shielded FP-LWAs utilize this TE_10_ mode to generate broadside radiation. However, as demonstrated by ref. 29, the aperture illumination efficiency of this mode is inherently limited to 81%, due to the non-optimal cosine-shaped aperture illumination^22^,^23^,^24^,^25^,^26^,^27^,^28^, leading to broadening of the main beam (inset of Fig. 2f). This highlights a key benefit of using an HMS-based antenna, as it is clear from Fig. 2f that we can use high-order mode excitations, which provide a more uniform illumination of the aperture, for generating narrow broadside beams with enhanced directivities.

In fact, as the mode index *n* increases, the autocorrelation of equation (4) drives the second harmonic peaks outside the visible region of the spectrum (shaded region in Fig. 2b,e), funnelling all the HMS-radiated power to the broadside beam, subsequently increasing the overall directivity. This improvement in radiation properties can be explained using ordinary array theory. As seen from Fig. 2d, the peaks of the field profile generated by the *n*th mode on the HMS aperture form hot spots of radiating currents separated by a distance of *L*/*n*. The radiation from such an aperture profile would resemble the one of a uniform array with the same element separation. As known from established array theory, to avoid grating lobes the element separation should be smaller than a wavelength^10^. For an aperture length of *L*=*Nλ*, where *N* is an integer, the hot-spot separation satisfies this condition for mode indices *n*>*N*; specifically, for *N*=10 (Fig. 2), grating lobes would not be present in the radiation pattern for mode indices *n*>10. In agreement with this argument, Fig. 2f shows that for *n*=9 grating lobes still exist, while for the highest order fast mode *n*=19 they indeed vanish.

**Figure 3:**
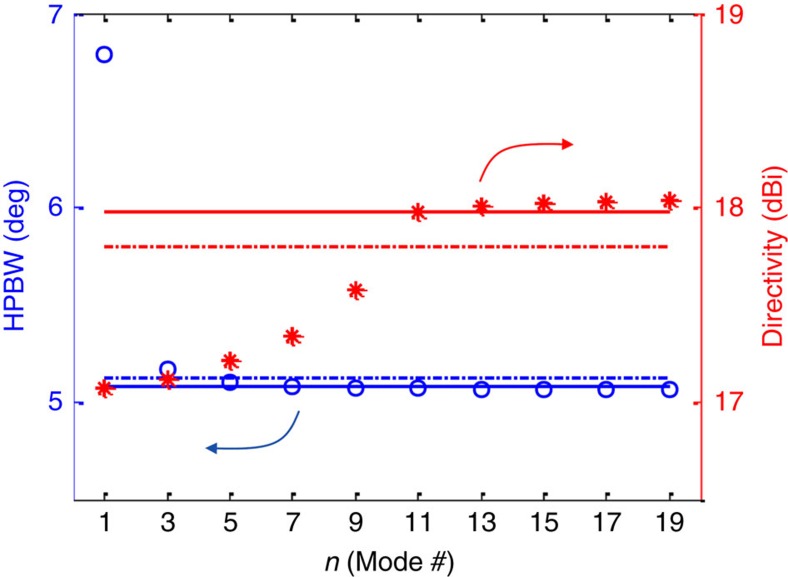
Radiation characteristics of different lateral cavity modes. Half-power beamwidth (HPBW, blue circles) and 2D directivity^58^ (red asterisks) of an HMS aperture of length *L*=10*λ* excited by a single mode as a function of the mode index *n*. Solid lines denote the respective radiation characteristics of a uniformly excited aperture^29^ and dash-dotted lines mark the HPBW (blue) and directivity (red) of *multimode* excitation corresponding to the HMS antenna of Fig. 1 with *L*=10*λ*, *z*′=−*λ*, and *d*=1.61*λ*.

These observations are summarized in Fig. 3, where the radiation characteristics of an HMS aperture of *L*=10*λ* excited by a single mode are plotted as a function of the mode index *n* (only fast modes *k*_t,*n*_=*nπ*/*L*<*k* are considered). Indeed, it can be seen that the lowest order lateral mode exhibits the worst performance, and the performance improves as the mode index increases. While the half-power beamwidth (HPBW) saturates quickly, the directivity *D* continues to increase with *n* until the point in which grating lobes disappear *n*=*N*=10 is crossed; for mode indices *n*>10 the radiation characteristics of the HMS aperture are comparable to those of the optimal uniformly excited aperture (solid lines).

From an array theory point of view, excitation of the highest order fast mode is preferable, as the corresponding equivalent element separation approaches *λ*/2, implying that such aperture profile would be suitable for directing the radiation to large oblique angles *θ*_out_≠0 without generating grating lobes^10^. Furthermore, as the HMS reflection coefficient Γ(*k*_t_) grows larger with *k*_t_=*nπ*/*L* (equation (3)), the power carried by the highest order fast-mode *n*=2*N*−1 is best-trapped in the cavity, guaranteeing uniform illumination even in the case of very large apertures.

Nevertheless, generating a single-mode excitation of a cavity via a localized source can be very problematic^30^,^45^. Fortunately, the cavity-excited HMS antenna can function very well also with multimode excitation, as long as high-order modes dominate the transmission spectrum. This is demonstrated by the dot-dashed lines in Figs 2 and 3, corresponding to a multimode excitation generated by the configuration depicted in Fig. 1 with *L*=10*λ*, *z* ′=−*λ*, and *d*=1.61*λ*. As expected from the expression for the source spectrum (equation (6)), for a given aperture length *L*, the field just below the aperture due to a line source would be a superposition of lateral modes, the weights of which are determined by the particular source configuration, namely the cavity thickness *d* and source position *z* ′. The multimode transmission spectrum in Fig. 2b indicates that for the chosen parameter values, high-order modes (*k*_t_→±*k*) predominantly populate the aperture spectrum, however, low-order modes (*k*_t_→0) are present as well, to a non-negligible extent. Considering that the far-field angular power distribution *S*(*θ*) is proportional to 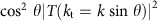, the multimode excitation of the PRS aperture results in a radiation pattern resembling the one corresponding to single-mode excitation of the highest order fast-mode (*n*=19) but with significant lobes around broadside (Fig. 2c); consequently, the directivity is significantly deteriorated.

On the other hand, the same multimode excitation does not degrade substantially the performance of the HMS antenna. The autocorrelated spectrum results in merging of all spectral components into a sharp d.c. peak, with most grating lobes pushed to the evanescent region of the spectrum (Fig. 2e). This retains a beamwidth comparable to that resulting from a single-mode excitation of the highest order fast mode, with only slight deterioration of the directivity due to increased side-lobe level (Fig. 2f and inset). Continuing the analogy to array theory, such multimode excitation introduces slight variations to the magnitude of the array elements, forming an equivalent non-uniform array^10^. The corresponding multimode HPBW and directivity values marked by dash-dotted lines in Fig. 3 verify that, indeed, cavity-excited HMS antennas achieve near-unity aperture illumination efficiencies with a practical multimode excitation; this points out another key advantage of the cavity-excited HMS antenna with respect to shielded FP-LWAs.

We utilize these observations to formulate guidelines for optimizing the cavity excitation for maximal directivity. For a given aperture length *L*=*Nλ*, with respect to equation (6), we maximize the coupling to the *n*=2*N*−1 mode (which exhibits the best directivity) by tuning the cavity thickness *d* as to minimize the denominator of the corresponding coupling coefficient; equally important, we minimize the coupling to the *n*=1 mode (which exhibits the worst directivity) by tuning the source position *z* ′ as to minimize the numerator of the corresponding coupling coefficient. To achieve these with minimal device thickness we derive the following design rules


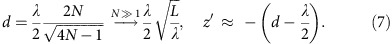


Although this is somewhat analogous to the typical design rules for FP-LWAs^13^, the key difference is that for HMS-based antennas we optimize the source configuration regardless of the desirable transmission angle *θ*_out_. This difference is directly related to the utilization of the equivalence principle for the design of the proposed device, which provides certain decoupling between its excitation and radiation spectra (cf. Fig. 2b,e). This decoupling becomes very apparent when the HMS antenna is designed to radiate towards oblique angles *θ*_out_≠0, in which case the same cavity excitation yields optimal directivity as well (see Supplementary Methods, Supplementary Fig. 1, and Supplementary Table 1).

Two important comments are relevant when considering these design rules. First, even though following equation (7) maximizes the coupling coefficient of the highest order fast mode and minimizes the coupling coefficient of the lowest order mode, it does not prohibit coupling to other modes. The particular superposition of lateral modes exhibits a tradeoff between beamwidth and side-lobe level (as for non-uniform arrays^10^). Thus, final semianalytical optimization of the cavity illumination profile is achieved by fine tuning the source position *z* ′ for the cavity thickness *d* derived in equation (7). In fact, the source position *z* ′ is another degree of freedom that can be used to optimize the radiation pattern for other desirable performance features, such as minimal side-lobe level; this feature is further discussed in the Supplementary Methods and demonstrated in Supplementary Figs. 2 and 3, and Supplementary Table 2. Second, although the optimal device thickness increases with increasing aperture length, the increase is sublinear. Therefore, applying the proposed concept to very large apertures would still result in a relatively compact device, while efficiently utilizing the aperture for producing highly directive beams.

### Physical implementation and radiation characteristics

We follow the design procedure and considerations discussed above to design cavity-excited HMS antennas for broadside radiation with different aperture lengths: *L*=10*λ*, *L*=14*λ*, and *L*=25*λ*. The cavity thickness was determined via equation (7) to be *d*=1.61*λ*, *d*=1.89*λ* and *d*=2.50*λ*, respectively; the source position was set to *z*′=−1.00*λ*, *z*′=−1.33*λ*, and *z*′=−1.94*λ*, respectively, exhibiting maximal directivity.

**Figure 4:**
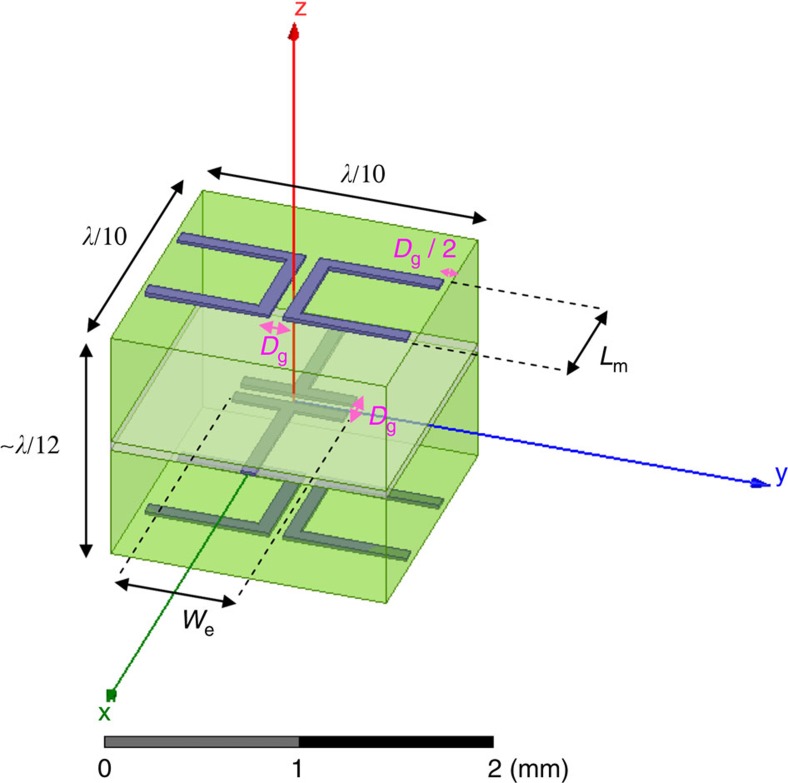
Spider unit cells. Physical configuration of the meta-atoms used for implementing the HMS at a frequency of *f*=20 GHz (*λ*≈15 mm). The electric response is controlled by the capacitor width *W*_e_ of the electric dipole, while the magnetic response is determined by the magnetic dipole arm length *L*_m_. The gap size (magenta) and copper trace width are fixed to *D*_g_=3 mil≈76 μm to comply with standard printed-circuit board fabrication techniques.

The required electric surface impedance and magnetic surface admittance modulations are implemented using the ‘spider' unit cells depicted in Fig. 4. At the design frequency *f*=20 GHz (*λ*≈15 mm), the unit cell transverse dimensions are *λ*/10 × *λ*/10 and the longitudinal thickness is 52 mil≈*λ*/12. Each unit cell consists of three layers of metal traces defined on two bonded laminates of high-dielectric-constant substrate (see Methods). The two (identical) external layers provide the magnetic response of the unit cell, corresponding to the magnetic surface susceptance *B*_sm_=ℑ{*Y*_sm_}, which is tuned by modifying the arm length *L*_m_ (affects equivalent magnetic currents induced by tangential magnetic fields *H*_*y*_). Analogously, the middle layer is responsible for the electric response of the meta-atom, corresponding to the electric surface reactance *X*_se_=ℑ{*Z*_se_}, which is tuned by modifying the capacitor width *W*_e_ (affects electric currents induced by tangential electric fields *E*_*x*_). By controlling *L*_m_ and *W*_e_, these unit cells can be designed to exhibit Huygens source behaviour, with balanced electric and magnetic responses ranging from *B*_sm_*η*=*X*_se_/*η*=−3.1 to *B*_sm_*η*=*X*_se_/*η*=0.9 (see Methods and Supplementary Fig. 4).

**Figure 5:**
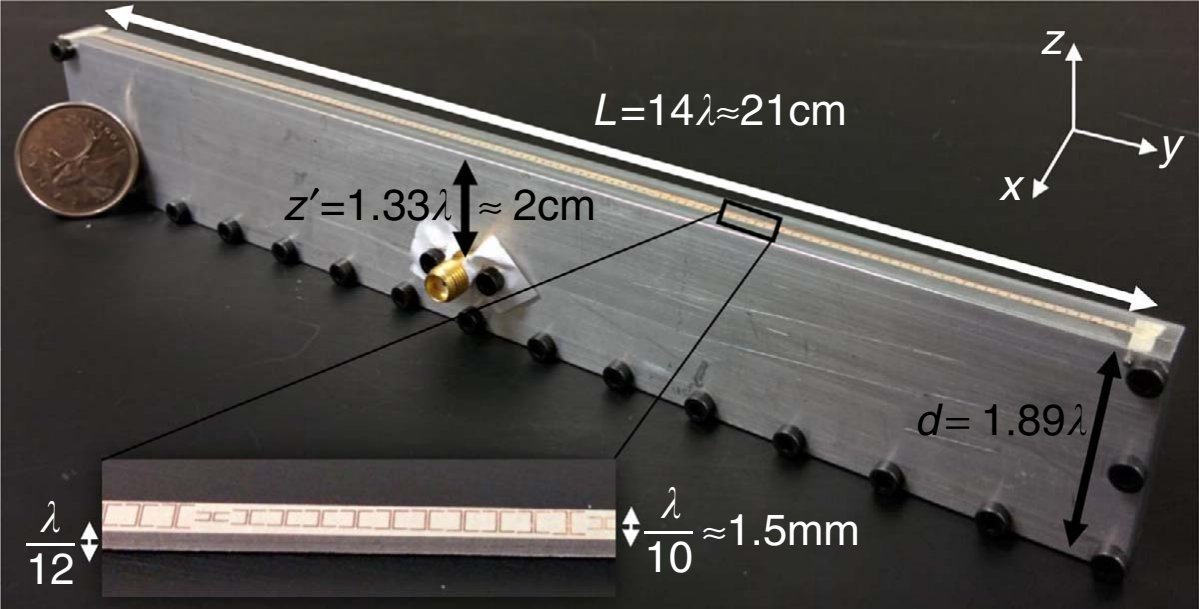
Fabricated cavity-excited HMS antenna. Probe-fed cavity excites a *λ*/10-wide *L*=14*λ*-long metastrip implementing the simulated design corresponding to Fig. 6b, based on the spider unit cells. The two metallic walls parallel to the 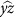 plane form a 2D excitation environment, while the two (shorter) metallic walls parallel to the 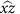 plane form the lateral cavity. Inset: close-up of a section of the metastrip before integration with the cavity.

To experimentally verify our theory, we have fabricated and characterized the *L*=14*λ* cavity-excited HMS antenna, based on the simulated spider cell design at *f*=20 GHz (see Methods). The antenna is composed of a one unit-cell-wide metastrip excited by a coaxial-cable-fed short dipole positioned inside an Aluminium cavity, forming the suitable 2D excitation configuration (Fig. 5). The aperture fields were allowed to radiate into (3D) free-space; the far-field radiation measured in the 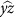 plane then corresponds to the theoretically predicted 2D radiation patterns.

**Figure 6:**
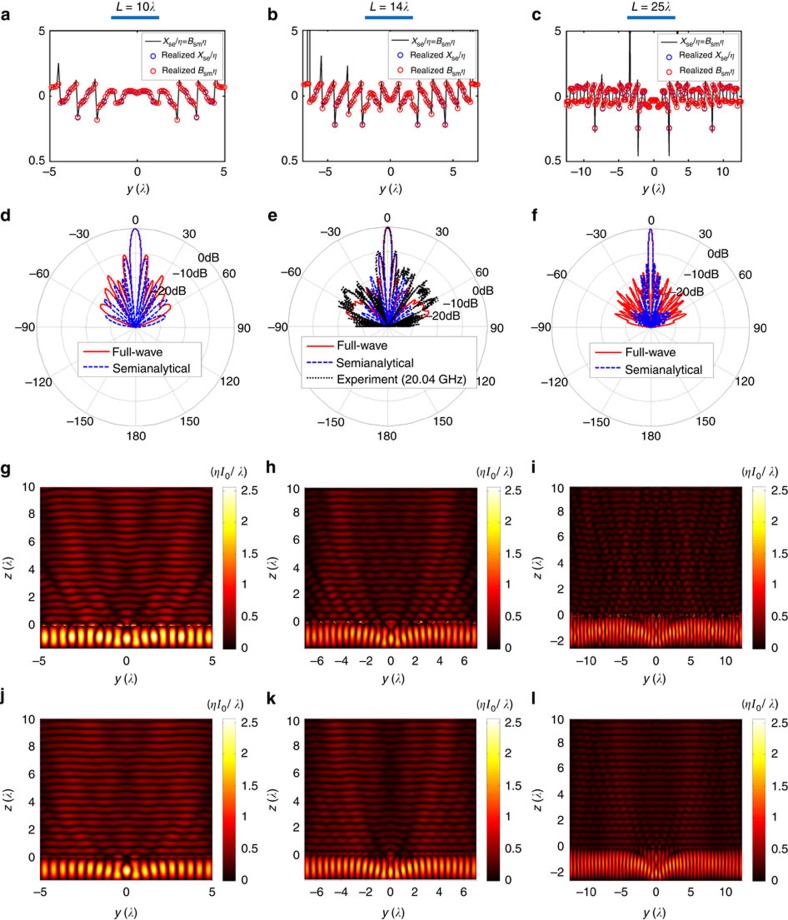
Performance of cavity-excited HMS antennas. Results are presented for devices with aperture lengths of *L*=10*λ*, *L*=14*λ*, and *L*=25*λ*. (**a**–**c**) HMS design specifications *X*_se_(*y*)/*η*=*B*_sm_(*y*)*η* (black solid line) derived from equation (5), and the realized electric surface reactance (blue circles) and magnetic surface susceptance (red circles) using the spider unit cells. (**d**–**f**) Radiation patterns produced by semianalytical formalism (blue dashed line) and full-wave simulations (red solid line). For *L*=14*λ*, the experimentally obtained radiation pattern is presented in black dotted line. (**g**–**i**) Field distribution 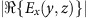 produced by full-wave simulations. (**j**–**l**) Semianalytical prediction of 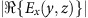 (ref. 40).

Figure 6 presents the design specifications, field distributions, and radiation patterns for the three cavity-excited HMS antennas; Table 1 summarizes the antenna performance parameters (for reference, parameters for uniformly excited apertures^29^ are also included). The semianalytical predictions^40^ are compared with full-wave simulations conducted with commercially available finite-element solver (ANSYS HFSS), as well as to experimental measurements where applicable (see Methods). As demonstrated by Fig. 6a–c, the realized unit cells are capable of reproducing the required surface impedance modulation, except maybe around large values of *B*_sm_*η*=*X*_se_/*η*; however, such discrepancies usually have little effect on the performance of HMSs^46^.

The results in Fig. 6 and Table 1 indicate that the fields and radiation properties predicted by the semianalyical formalism are in excellent agreement with the full-wave simulations for a wide range of aperture lengths. The utilization of realistic (lossy) models for the conductors and dielectrics in the simulated device, as well as other deviations from the assumptions of the design procedure (Supplementary Note 1), result in some discrepancies between the full-wave simulations and predicted performance; however, these mostly affect radiation to large angles (Fig. 6d–f). While this contributes to a minor quantitative difference in the directivity, the properties of the main beam and the side lobes follow accurately the semianalytical results (Table 1), indicating that the theory can reliably predict the dominant contributions to the radiation pattern, as discussed in reference to Fig. 2.

This conclusion is further supported by the experimental results presented for the *L*=14*λ* antenna at *f*=20.04 GHz, where good agreement between theoretical and measured radiation patterns is observed (Fig. 6e). The experimental values of the HPBW, directivity and side-lobe level and position documented in Table 1 also agree quite well with the simulated ones. The slightly higher side-lobe levels and the broadening of the side lobe at *θ*=−7.2° contribute to a smaller measured directivity value, and can be attributed to fabrication errors. Nevertheless, the fact that the main features of the radiation pattern are reproduced well with only negligible deviation of 0.2% from the design frequency, and the fact that the predicted, simulated and measured main beams practically coincide, forms a solid validation of our theory.

The measured frequency response of the antenna presented in Supplementary Fig. 5a also compares very well with the simulated results, indicating a measured 3- and 1-dB directivity bandwidths of 5.5% and 1.3%, respectively. These values are not very high, but this is expected due to the resonant nature of the cavity and metasurface^24^,^38^. Bandwidth enhancement can be likely achieved by using stacked HMSs or increasing the number of layers comprising the unit cells^24^,^47^. The measured 10-dB return-loss bandwidth is 0.2% (Supplementary Fig. 5b), comparable to the values reported for cavity-based antennas^48^; nonetheless, it could be improved by using suitable matching circuitry^49^. The measured (3D) realized gain at *f*=20.04 GHz is 16.12±1 dBi, corresponding to a gain of 17.21±1 dBi (with 2.7% 3-dB bandwidth). From the 3D directivity estimated from the measurements (Supplementary Methods and Supplementary Fig. 6) we evaluate a radiation efficiency of 75±17%, in a reasonable agreement with the ≈15% conductor and dielectric losses predicted by full-wave simulations (Supplementary Fig. 7). These results indicate that if impedance matching can be achieved via suitable circuitry, then cavity-excited HMS antennas could exhibit reasonably high antenna efficiencies. Lastly, the measured cross-polarization gain was below the noise floor, indicating at least 30-dB cross-polar discrimination at broadside.

For all cases considered, the excitation of the highest order lateral fast mode is clearly visible (Fig. 6g–l), leading to beamwidth and (2D) directivity values comparable to the ones achieved by uniform excitation of the aperture (Table 1). In particular, the simulated (measured) radiation patterns yield aperture illumination efficiencies 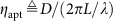 of 88%, 86% (75%) and 87% for the *L*=10*λ*, *L*=14*λ* and *L*=25*λ* devices, respectively, retained even when the aperture length is very large. In terms of HPBW, often taken as a measure for effective exploitation of the aperture^19^, the device performance is even closer to that of a unifromly excited aperture, with pencil-beam HPBWs reaching 99%, 95% (100%) and 97% of the optimal beamwidth, for *L*=10*λ*, *L*=14*λ* and *L*=25*λ* devices, respectively. Similar to FP-LWAs^13^, the simulated 3-dB bandwidths of the antennas reveal a tradeoff between directivity and bandwidth, reflected by an approximately constant value of 3.1 for the directivity-bandwidth product (Supplementary Fig. 8).

It should be stressed that even though optimized TE_10_ shielded FP-LWAs can reach aperture illumination efficiencies of 81%, their HPBWs are limited to about 75% of the optimal beamwidth^29^; more importantly, their PRS-based design requires single-mode excitation to achieve this performance, thus preventing practical realization of large-aperture devices. Just recently, a low-profile metasurface-based lens antenna with a smaller aperture length (diameter of 6.6*λ*) operating at a lower frequency (10 GHz), has been reported^50^, also exhibiting a high aperture illumination efficiency. Nevertheless, this antenna utilizes a metasurface designed to convert Bessel beams to Gaussian beams; hence, to excite it, a Bessel beam launcher has to be separately designed and fabricated for each application, in addition to the metasurface lens. In contrast, our design approach and rationale can be straightforwardly applied to apertures of any size, and yields an integrated air-filled device requiring only a single simple feed.

While the main purpose of the proposed solution is to maximize antenna directivity, this design goal is suitable in cases where the environment clatter is relatively controlled (for example, point-to-point communications^4^), as uniformly illuminated apertures incur relatively high side-lobe levels; for many applications lower side-lobe levels are required^7^. Nonetheless, as discussed in the Supplementary Methods and demonstrated in Supplementary Fig. 3, a desirable compromise between directivity and side-lobe level can be achieved in the framework of our theory, by simple tuning of the source position *z* ′. Setting the cavity thickness *d* following equation (7) still forms a virtual antenna array with linear phase, while the different values of *z* ′ effectively vary the magnitude of the array elements, facilitating side-lobe control^10^. The example presented in Supplementary Fig. 2 and Supplementary Table 2 harnesses this scheme to design (*L*=10*λ*)-long cavity-excited HMS antennas with side-lobe level of −20 dB, still retaining a rather high aperture illumination efficiency of 81%.

## Discussion

We have introduced a novel design for low-profile single-fed antennas exhibiting beamwidth and directivity values comparable with uniformly excited apertures. To that end, we harness the equivalence principle to devise a cavity-excited Huygens' metasurface, setting the source configuration, HMS reflection coefficient, and aperture fields such that (1) the highest order (lateral) fast mode is predominantly excited, which guarantees that the aperture is well-illuminated; (2) the aperture fields follow the incident power profile and not the incident field profile, which forms an array-like aperture profile with favourable transmission spectrum; and (3) the power flow and wave impedance are continuous across the metasurface, which ensures the design can be implemented by a passive and lossless HMS^40^. The possibility to control the field discontinuities using the electric and (equivalent) magnetic currents induced on the HMS allows us to optimize separately the cavity excitation and the radiated fields, thus overcoming the fundamental tradeoff existing in FP-LWAs between aperture illumination efficiency and edge-taper losses.

It should be emphasized that the general design procedure formulated and demonstrated herein facilitates further optimization of such devices for various applications. The extensive freedom one has in choosing the source configuration, combined with the efficient semianalytical approach, allows explorations of other excitation sources, for example, with different orientations and current distributions, to further tailor the aperture fields (extension to 3D configurations and polarization control are discussed in Supplementary Note 2); once the source spectrum is characterized, the rest of the procedure is straightforward, and the fields and radiation patterns are readily predicted. In addition, considering the recent demonstrations of metasurfaces in general and Huygens' metasurfaces in particular, operating at terahertz and optical frequencies^36^,^39^,^51^,^52^,^53^,^54^,^55^,^56^, the proposed methodology could be applied to realize compact and efficient pencil beam radiators across the electromagnetic spectrum, extending the range of applications even further.

## Methods

### Spider unit-cell modelling

The spider unit cells depicted in Fig. 4 were defined in ANSYS Electromagnetic Suite 15.0 (HFSS 2014) with two 25-mil-thick (≈0.64 mm) Rogers RT/duroid 6010LM laminates (green boxes in Fig. 4) bonded by 2-mil-thick (≈51 μm) Rogers 2929 bondply (white box in Fig. 4). The electromagnetic properties of these products at 20 GHz, namely, permittivity tensor and dielectric loss tangent, as were provided to us by Rogers Corporation, have been inserted to the model. Specifically, a uniaxial permittivity tensor with *ɛ*_*xx*_=*ɛ*_*yy*_=13.3*ɛ*_0_, *ɛ*_*zz*_=10.81*ɛ*_0_ and loss tangent of tan *δ*=0.0023 were considered for Rogers RT/duroid 6010LM laminates, while an isotropic permittivity of *ɛ*=2.94*ɛ*_0_ and loss tangent tan*δ*=0.003 were considered for Rogers 2929 bondply. The copper traces corresponded to 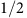 oz. cladding, featuring a thickness of 18 μm; the standard value of *σ*=58 × 10^6^ S m^−1^ bulk conductivity was used in the model. To comply with standard printed-circuit board manufacturing processes, all copper traces were 3 mil (≈76 *μ*m) wide, and a minimal distance of 3 mil was kept between adjacent traces (within the cell or between adjacent cells). This implies that the fixed gaps between the capacitor traces (along the *x* axis) of the electric dipole in the middle layer, as well as between the two arms (along the *y* axis) of the magnetic dipole in the top and bottom layer (Fig. 4), were fixed to a value of *D*_g_=3 mil (≈76 *μ*m); the distance from the arm edge to the edge of the unit cell was fixed to *D*_g_/2=1.5 mil (≈38 *μ*m).

Unit cells with different values of magnetic dipole arm length *L*_m_ and electric dipole capacitor width *W*_e_ were simulated using periodic boundary conditions; HFSS Floquet ports were placed at *z*=±*λ* and used to characterize the scattering of a normally incident plane wave off the periodic structure (the interface between the bondply and the bottom laminate was defined as the *z*=0 plane). For each combination of *L*_m_ and *W*_e_, the corresponding magnetic surface susceptance *B*_sm_ and electric surface reactance *X*_se_ were extracted from the simulated impedance matrix of this two-port configuration, following the derivation in ref. 57.

The magnetic response *B*_sm_ was found to be proportional to the magnetic dipole arm length *L*_m_, with almost no dependency in *W*_e_ (ref. 37). Thus, to create an adequate lookup table for implementing broadside-radiating HMSs, we varied *L*_m_ by constant increments, and for a given *L*_m_, plotted *B*_sm_*η* and *X*_se_/*η* as a function of *W*_e_. The value of *W*_e_ for which the two curves intersected corresponded to a balanced-impedance point (*Z*_se_/*Z*_out_=*Y*_sm_/*Y*_out_), where the unit cell acts as a Huygens source, and thus suitable for implementing our metasurface. A lookup table composed of (*B*_sm_, *X*_se_) pairs and the corresponding unit cell geometries (*L*_m_, *W*_e_) was constructed, and refined through interpolation. The interpolated unit cell geometries were eventually simulated again, to verify the interpolation accuracy and finalize the lookup table entries, as presented in Supplementary Fig. 4. Finally, for a given HMS with prescribed surface impedance modulation (*B*_sm_(*y*), *X*_se_(*y*)), a corresponding structure could be defined in HFSS using the unit cells (*L*_m_(*y*), *W*_e_(*y*)) found via the lookup table in terms of least squares error.

### Antenna simulations

To verify our semianalytical design via full-wave simulations, each of the cavity-excited HMS antennas designed in this paper was defined in HFSS using a single strip of unit cells implementing the metasurface, occupying the region |*x*|≤*λ*/20, |*y*|≤*L*/2 (*L* being the aperture length of the antenna), and −0.64 mm≤*z*≤0.69 mm (in correspondence to the laminate and bondply thicknesses). The simulation domain included |*x*|≤*λ*/20, |*y*|≤*L*/2+2.5*λ*, and −*d*≤*z*≤10*λ* (*d* being the cavity thickness), where PEC boundary conditions were applied to the *x*=±*λ*/20 planes to form the equivalence of a 2D scenario. PEC boundary conditions were also applied to the *z*=−*d* plane, and to two 18-μ*m*-thick side-walls at *y*=±*L*/2, forming the cavity. The line-source excitation was modelled by a *λ*/20-wide 1A current sheet at *z*=*z*′, with the current aligned with the *x* axis. Radiation boundary conditions were applied to the rest of the simulation space boundaries, namely *z*=10*λ*, and *y*=±(*L*/2+2.5*λ*), allowing proper numerical evaluation of the fields surrounding the antenna.

To reduce the computational effort required to solve this configuration, we utilized the symmetries of our TE scenario. Specifically, we placed a perfect-magnetic-conductor (PMC) symmetry boundary conditions at the 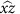 plane, and a PEC symmetry boundary conditions at the 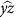 plane. We also noticed that adding a thin layer (1 mil≈25 μm) of copper between the electric dipole edges and the PEC parallel plates at *x*=±*λ*/20 enhanced the convergence of the simulation results. With that minor modification, all of the simulated antennas converged within <40 iterations (maximum refinement of 10% per pass), where the stop conditions was three consecutive iterations in which ΔEnergy<0.03.

### Antenna realization and measurement

The simulated design corresponding to the cavity-excited HMS antenna with *L*=14*λ* aperture (Fig. 6b) has been realized and measured to obtain experimental validation of our theory. As in the full-wave simulations, the fabricated metasurface consisted of two 25-mil-thick (≈0.64 mm) Rogers RT/duroid 6010LM laminates. The copper traces' geometry used in the simulations to implement the spider unit cells was exported to standard grbr files, later used by Candor Industries Inc. to accordingly etch the electrodeposited 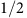 oz. copper foils covering the laminates. The etched laminates were then bonded using 2-mil-thick (≈51 *μ*m) Rogers 2929 bondply, forming the desirable three-layer metasurface. Lastly, unit-cell-wide (*λ*/10≈1.5 mm) metastrips were achieved via routing (see inset of Fig. 5).

The cavity was realized by replacing the PEC walls utilized in simulations by 4-mm-thick Aluminium. The resultant five-faceted box was split along the 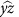 plane into two parts to enable accurate fabrication using computerized numerical control (CNC) machines in the University of Toronto; the two parts of the box were attached using multiple metallic screws along the box perimeter (Fig. 5). A subminiature version A (SMA) female connector flange with an exposed pin was mounted using screws on the front facet to feed the current source exciting the structure. The current source was created by extending the exposed pin using a soldered copper wire, forming an electric dipole with an overall length of *λ*/10, in accordance to the simulated configuration.

Finally, the metastrip was vertically positioned using two dowel pins inserted at the edges of the cavity aperture, where the electric field is predicted to be negligible. When assembling the antenna, the horizontal alignment of the metastrip was insured by the use of masking tape strips while the box peripheral screws were tightened; the tape was removed before measurements were conducted. To guarantee good coupling between the middle layer copper traces (Fig. 4) and the metallic walls, a thin adhesive copper film was attached to the metallic walls where they made contact with the the metastrip facets.

Antenna measurements were conducted in the far-field measurement chamber at the University of Toronto, calibrated using Quinstar Technology Inc. QWH-KPRS00 standard-gain horn antennas following the gain comparison method. The HMS antenna was then mounted on a stage situated in the far field of a transmitting horn antenna, and radiation patterns were obtained by rotating the stage in steps of 0.2°. The received power was spectrally resolved with a frequency resolution of 0.02 GHz within the frequency range *f*∈[18.1, 21.9 GHz]. The procedure for evaluating the antenna gain, directivity, radiation efficiency, and aperture illumination efficiency out of the measured radiation patterns is addressed in detail in the Supplementary Methods.

## Additional information

**How to cite this article:** Epstein, A. *et al.* Cavity-excited Huygens' metasurface antennas for near-unity aperture illumination efficiency from arbitrarily large apertures. *Nat. Commun.* 7:10360 doi: 10.1038/ncomms10360 (2016).

## Supplementary Material

Supplementary Information Supplementary Figures 1–8, Supplementary Tables 1&2, Supplementary Notes 1&2, Supplementary Methods and Supplementary References.

## Figures and Tables

**Table 1:** Radiation characteristics of cavity-excited HMS antennas.

	***L*****=10*****λ*** **(*****d*****=1.61*****λ*****, |*****z*****′|=1.00*****λ*****)**			***L*****=14*****λ*** **(*****d*****=1.89*****λ*****, |*****z*****′|=1.33*****λ*****)**				***L*****=25*****λ*** **(*****d*****=2.50*****λ*****, |*****z*****′|=1.94*****λ*****)**		
	**Full-wave**	**Semianalytic**	**Uniform**	**Experiment**	**Full-wave**	**Semianalytic**	**Uniform**	**Full-wave**	**Semianalytic**	**Uniform**
HPBW	5.11°	5.13°	5.08°	3.63°	3.83°	3.64°	3.63°	2.09°	2.13°	2.03°
Directivity (2D) (dBi)	17.42	17.84	17.98	18.20	18.79	19.15	19.44	21.33	21.75	21.96
First side-lobe	8.6°	8.6°	8.2°	−7.2°|6.0°	6.4°	6.3°	5.9°	3.4°	3.4°	3.3°
Side-lobe level (dB)	−10.4	−12.0	−13.5	−9.1	−11.1	−10.4	−13.5	−14.6	−14.0	−13.5

HMS, Huygens' metasurface; HPBW, half-power beamwidth; 2D, two dimensional.
