# Supplementary material for: Cavity-excited Huygens' metasurface antennas for near-unity aperture illumination efficiency from arbitrarily large apertures
Source: Nat Commun. 2016 Jan 21;7:10360. doi: 10.1038/ncomms10360 (PMC4735857; doi:10.1038/ncomms10360)
Supplement: Supplementary Information — Supplementary Figures 1–8, Supplementary Tables 1&2, Supplementary Notes 1&2, Supplementary Methods and Supplementary References. [file ncomms10360-s1.pdf]

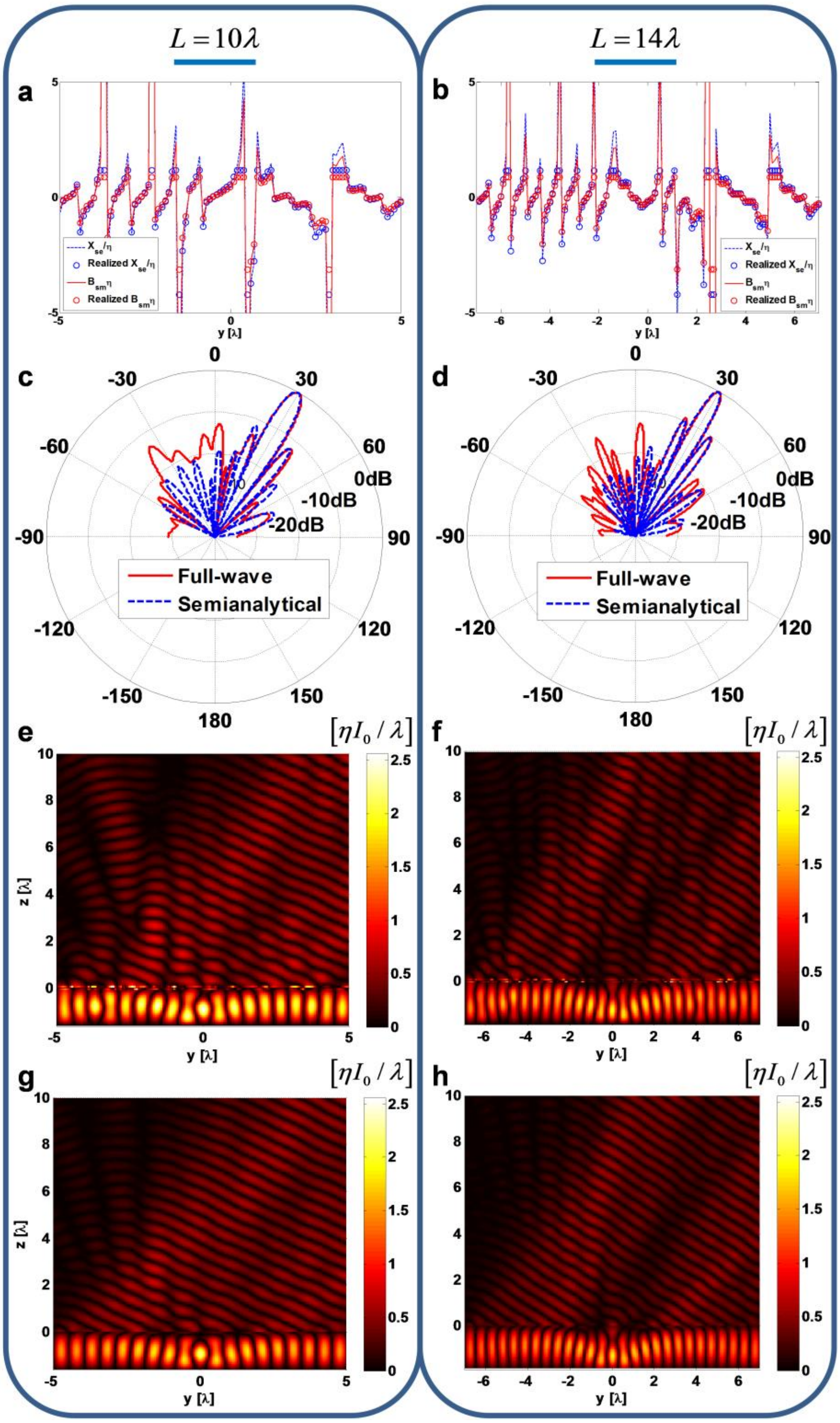


**Supplementary Figure 1 | Performance of cavity-excited HMS antennas radiating towards $\theta_{\text{out}} = 30°$.** Results are presented for devices with aperture lengths $L = 10\lambda$ and $L = 14\lambda$. (**a**,**b**) Specified (blue dashed line) and realized (blue open circles) electric surface reactance, and specified (red solid line) and realized (red open circles) magnetic surface susceptance using the spider unit-cells. (**c**,**d**) Radiation patterns produced by semianalytical formalism (blue dashed line) and full-wave simulations (red solid line). (**e**,**f**) Field distribution $\left|\Re\left\{E_x\left(y,z\right)\right\}\right|$ from full-wave simulations. (**g**,**h**) Semianalytical prediction of $\left|\Re\left\{E_x\left(y,z\right)\right\}\right|$.

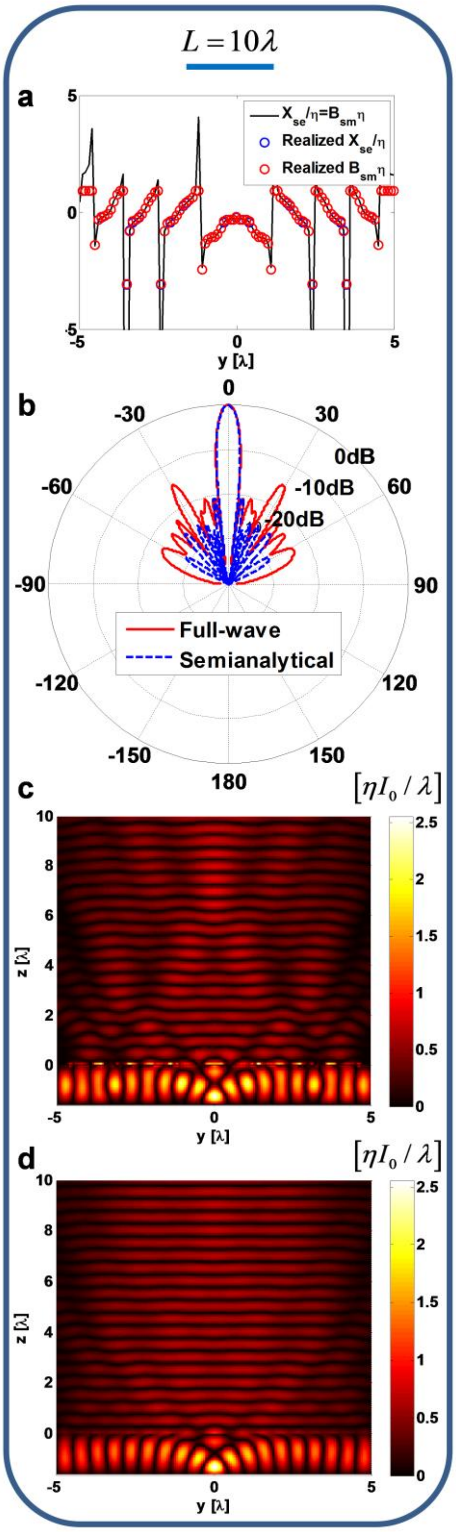


**Supplementary Figure 2 | Performance of a cavity-excited HMS with reduced side-lobe level.** Results are presented for a device with aperture length $L = 10\lambda$. (**a**) HMS design specifications $X_{\text{se}}(y)/\eta = B_{\text{sm}}(y)\eta$ (black solid line), and the realized electric surface reactance (blue open circles) and magnetic surface suseptance (red open circles) using the spider unit-cells. (**b**) Radiation patterns produced by semianalytical formalism (blue dashed line) and full-wave simulations (red solid line). (**c**) Field distribution $\left|\Re\left\{E_x(y,z)\right\}\right|$ from full-wave simulations. (**d**) Semianalytical prediction of $\left|\Re\left\{E_x(y,z)\right\}\right|$.

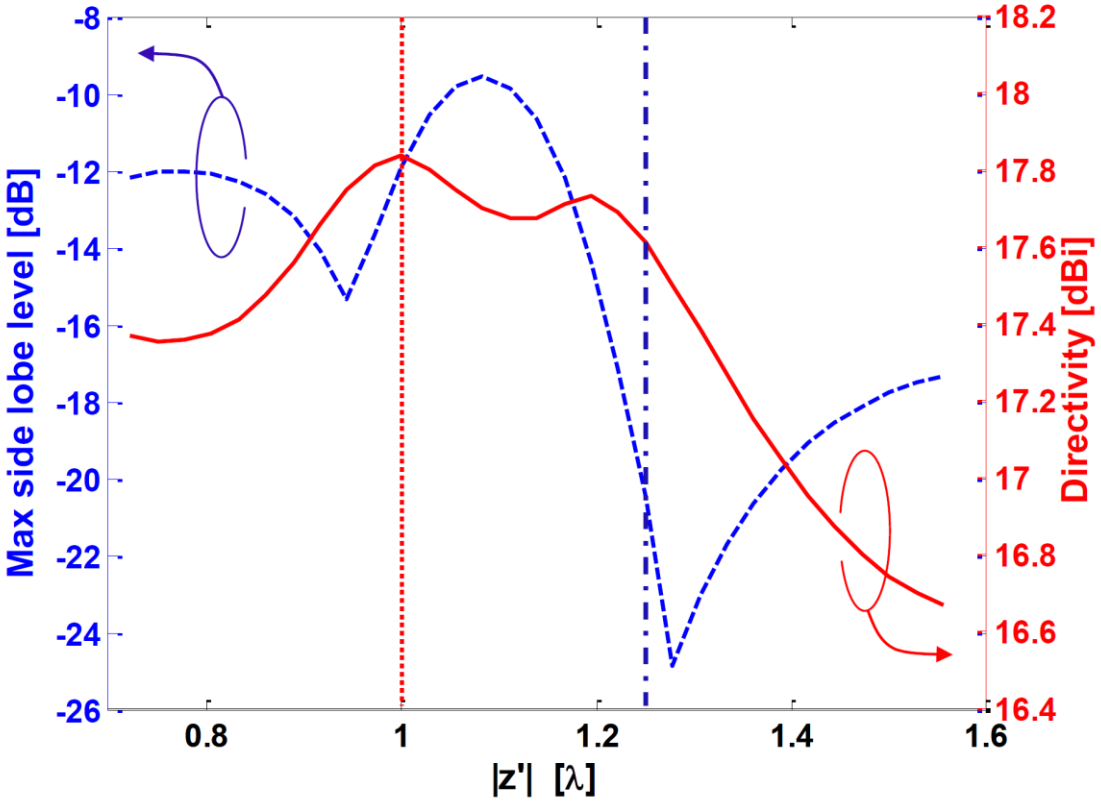


**Supplementary Figure 3 | Utilizing the source position as an additional degree of freedom for achieving desirable radiation characteristics.** 2D Directivity (red solid line) and maximal side-lobe level (dashed blue line) of a cavity-excited HMS antenna with $L = 10\lambda$ and $d = 1.61\lambda$ are presented as a function of source position $|z'|$. The source position maximizing directivity ($|z'| = 1\lambda$, corresponding to Fig. 6a,d,g,j) is denoted by a red dotted line, and the source position for reduced side-lobe level is denoted by a blue dash-dotted line ($|z'| = 1.25\lambda$, corresponding to Supplementary Fig. 2).

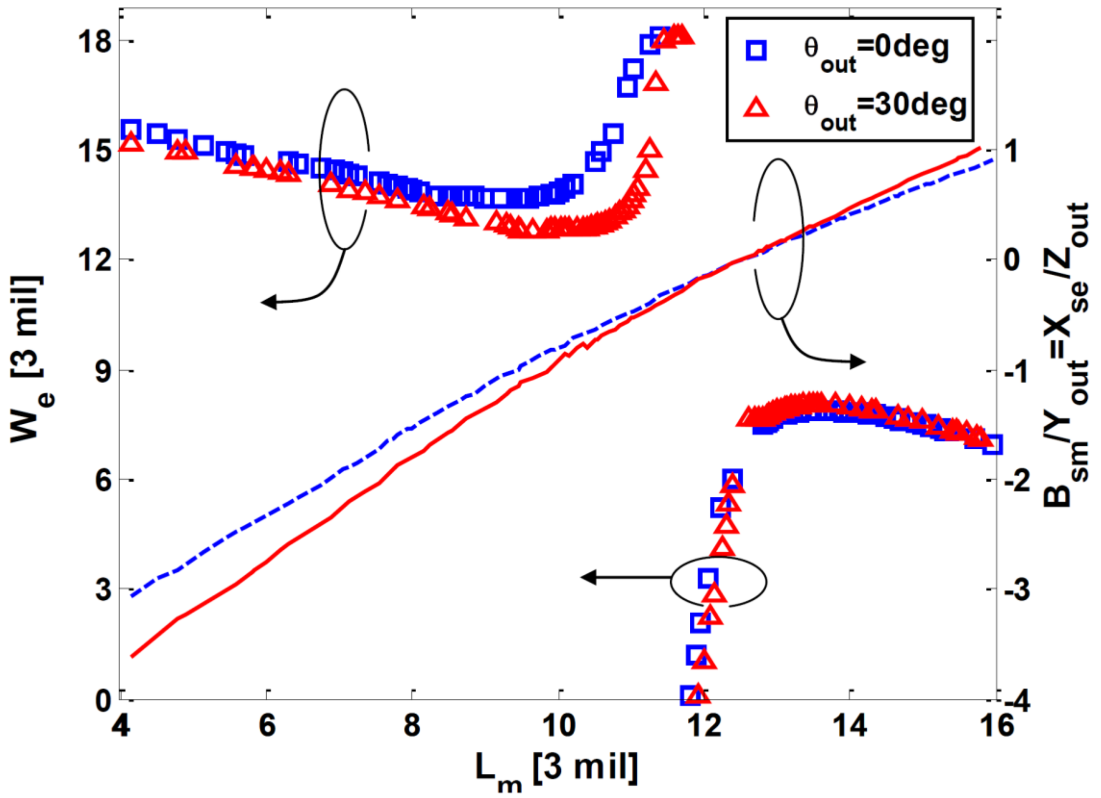


**Supplementary Figure 4 | Spider unit-cell lookup tables.** Graphic representation of tables corresponding to HMSs radiating towards $\theta_{\text{out}} = 0°$ and $\theta_{\text{out}} = 30°$ are presented. Capacitor width values $W_{\text{e}}$ required for achieving balanced electric and magnetic responses $B_{\text{sm}} / Y_{\text{out}} = X_{\text{se}} / Z_{\text{out}}$ are presented as a function of the magnetic dipole arm length $L_{\text{m}}$ for $\theta_{\text{out}} = 0°$ (blue open squares) and $\theta_{\text{out}} = 30°$ (red open triangles) radiators, as obtained by finite-element simulations (See Methods). The corresponding $B_{\text{sm}} / Y_{\text{out}} = X_{\text{se}} / Z_{\text{out}}$ values are denoted using a blue dashed line for $\theta_{\text{out}} = 0°$ ( $Z_{\text{out}} = 1/Y_{\text{out}} = \eta$ ) and using a red solid line for $\theta_{\text{out}} = 30°$ ( $Z_{\text{out}} = 1/Y_{\text{out}} = \eta / \cos 30°$ ). The plot and corresponding lookup table provide the suitable values of $(L_{\text{m}}, W_{\text{e}})$ for a spider unit cell locally implementing a given electric and magnetic responses $(B_{\text{sm}}, X_{\text{se}})$. The realizable range of surface impedance is limited by the unit cell dimensions $\lambda / 10 \approx 59\text{mil}$ and the minimal trace spacing requirements.

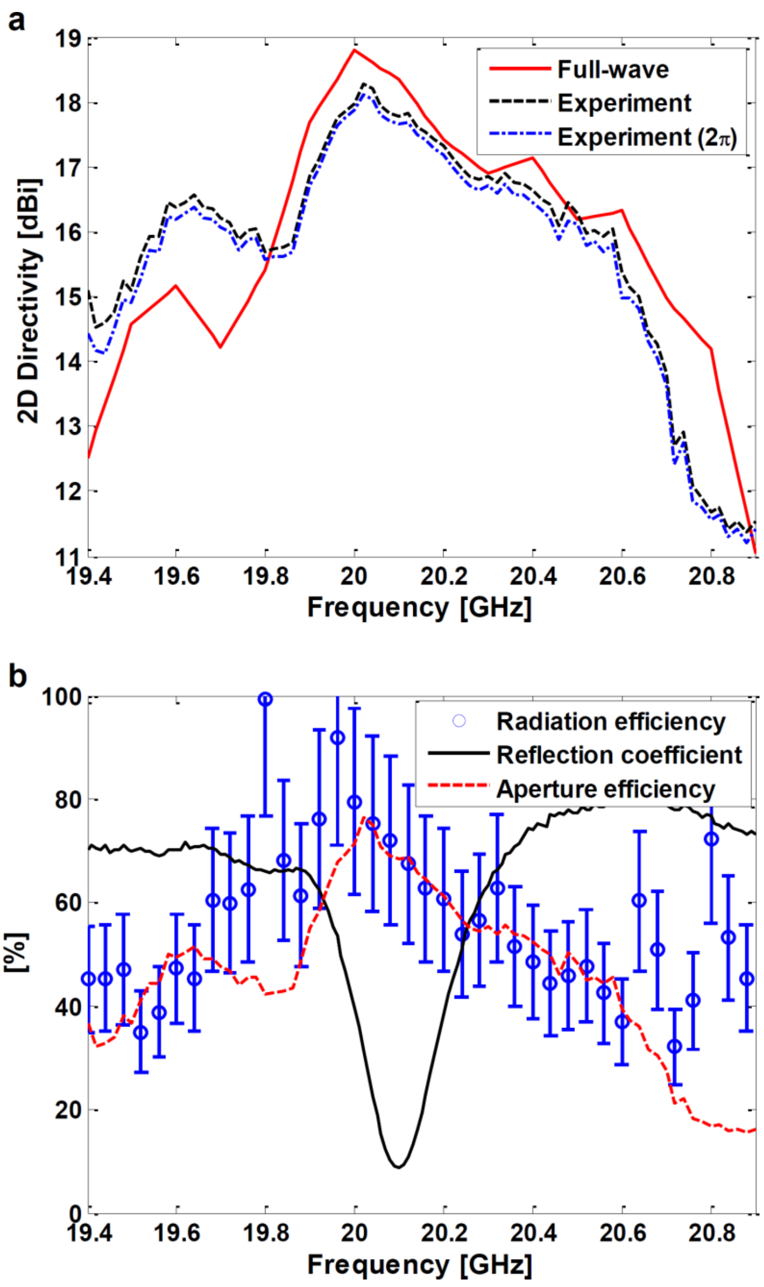


**Supplementary Figure 5 | Frequency response of the measured antenna.** Results are shown for the antenna with $L = 14\lambda$, designed following Fig. 6b, and experimentally characterized in Fig. 6e and Table I. (**a**) Comparison of simulated (red solid line) and measured (black dashed line) 2D directivity $D$ as a function of frequency. For completeness, the measured directivity considering backwards radiation $D_{2\pi}$ is also presented in blue dot-dashed line (see Supplementary Methods). (**b**) Measured aperture illumination efficiency $\eta_{\text{apt}} = D / (2\pi L / \lambda)$ (red dashed line), radiation efficiency $\eta_{\text{rad}} = G_{\text{3D}} / D_{\text{3D}}$ (blue circles) calculated from the 3D gain $G_{\text{3D}}$ and directivity $D_{\text{3D}}$, and (power) reflection coefficient $R$ (black solid line), as a function of frequency. See Supplementary Methods for detailed information regarding the evaluation procedure for $\eta_{\text{apt}}$, $G_{\text{3D}}$, $D_{\text{3D}}$, $\eta_{\text{rad}}$ and corresponding error bars.

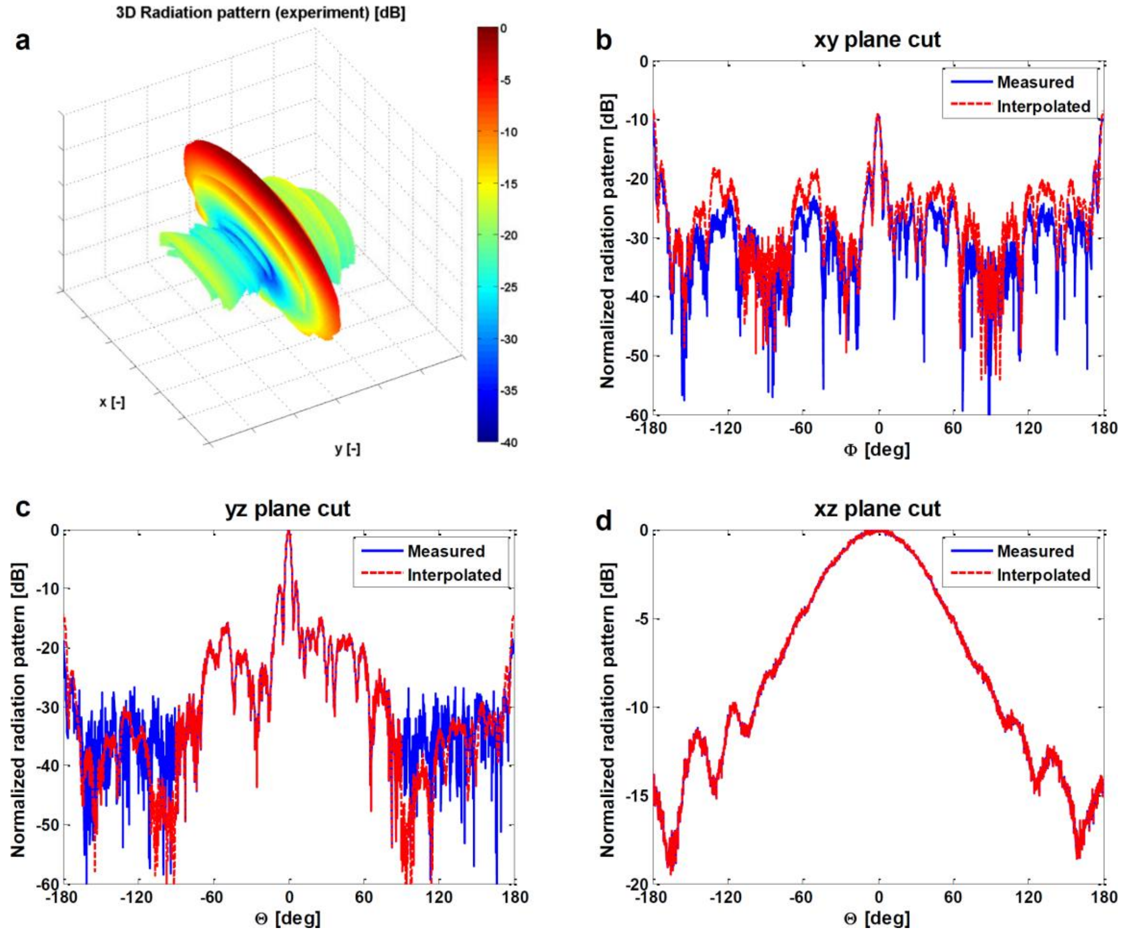


**Supplementary Figure 6 | Evaluation of experimental 3D radiation pattern.** Results are shown for the antenna with $L = 14\lambda$ at $f = 20.04\text{GHz}$, designed following Fig. 6b, and experimentally characterized in Fig. 6e and Table I. (**a**) 3D radiation pattern (dB scale) evaluated by interpolating measurements in the $yz$ and $xz$ plane cuts, as detailed in the Supplementary Methods. (**b**) Comparison between measured (blue solid line) and interpolated (red dashed line) radiation pattern in the $xy$ plane cut. (**c**) Comparison between measured (blue solid line) and interpolated (red dashed line) radiation pattern in the $yz$ plane cut. (**d**) Comparison between measured (blue solid line) and interpolated (red dashed line) radiation pattern in the $xz$ plane cut.

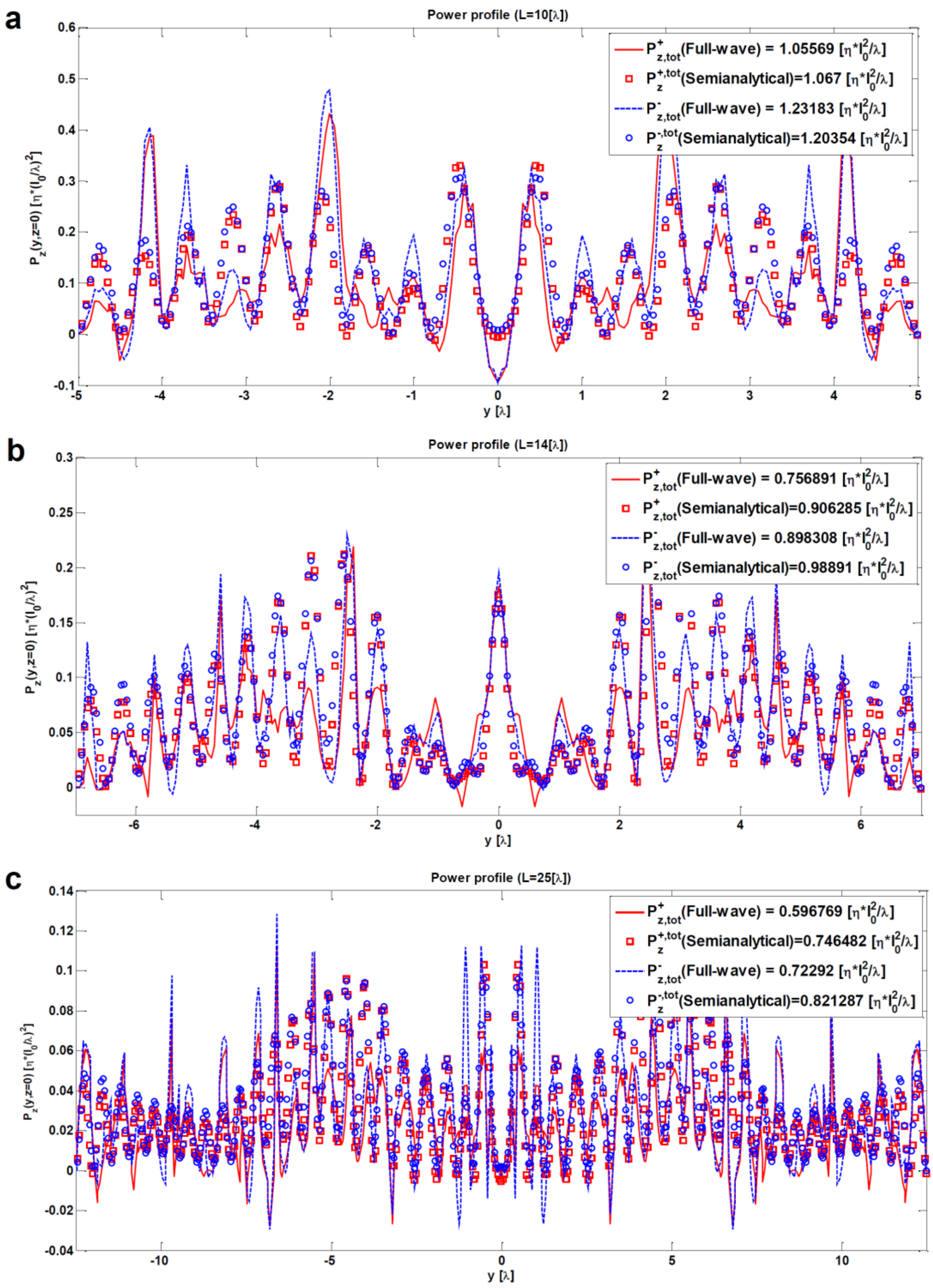


**Supplementary Figure 7 | Power profiles on the cavity-excited HMS antenna apertures.** The real part of the $z$-component of the Poynting vector $P_z^{\pm} = \Re\left\{S_z\left(y, z \to 0^{\pm}\right)\right\}$ above and below and the metasurface is presented for the antennas reported in Table I and Fig. 6, with aperture lengths (**a**) $L = 10\lambda$ (**b**) $L = 14\lambda$, and (**c**) $L = 25\lambda$. Full-wave simulation results of $P_z^{+}$ (red solid line) and $P_z^{-}$ (blue dashed line) were evaluated at $z = \lambda/10$ and $z = -\lambda/10$, respectively. Semianalytical predictions of $P_z^{+}$ (red open squares) and $P_z^{-}$ (blue open circles) are presented as well. The total power $P_{z,\text{tot}}^{\pm}$, calculated by integrating the power profile along the aperture is indicated in the legend.

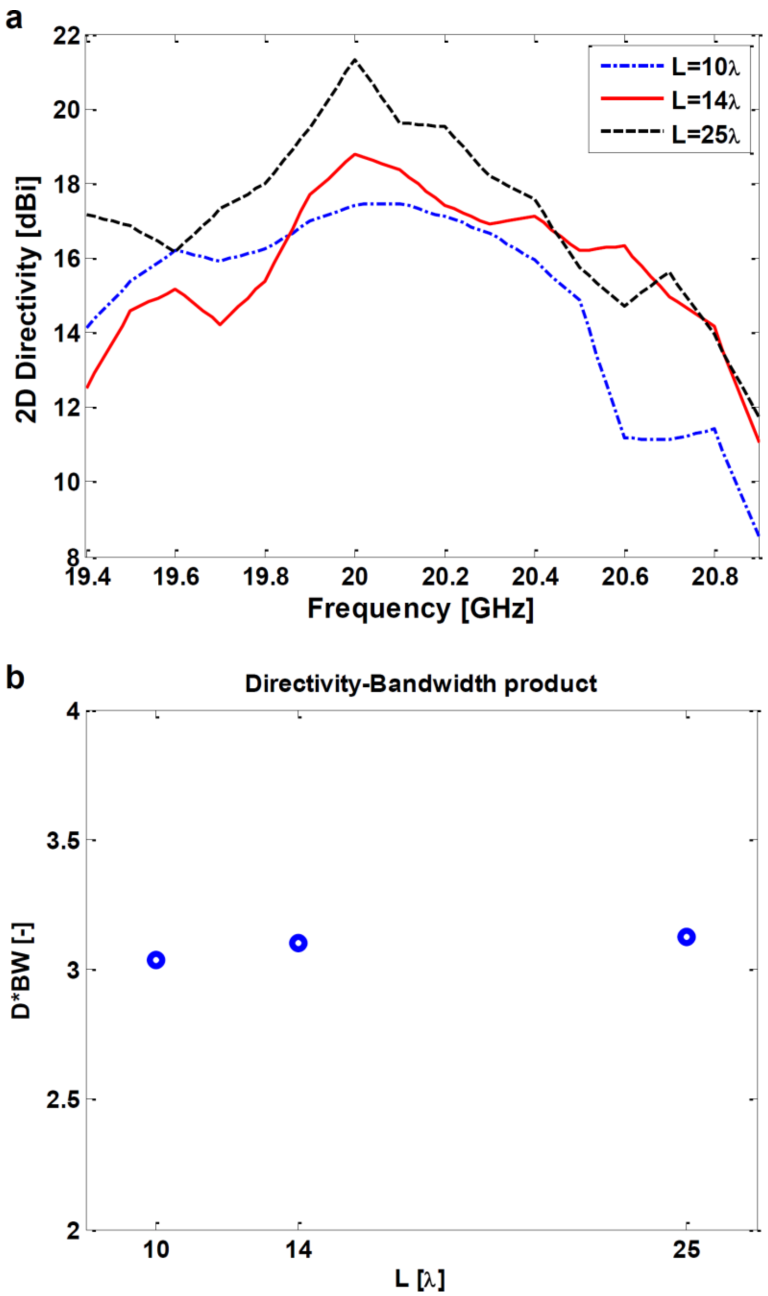


**Supplementary Figure 8 | Directivity-bandwidth characteristics of cavity-excited HMS antennas.** Results of full-wave simulations are shown for the antennas characterized in Fig. 6 and Table I. (**a**) Simulated 2D directivity of the antennas with aperture length with $L=10\lambda$ (blue dash-dotted line), $L=14\lambda$ (red solid line), and $L=25\lambda$ (black dashed line), exhibiting fractional bandwidths (BW) of 5.5%, 4.1%, and 2.3%, respectively. (**b**) Directivity-bandwidth product (D*BW) for the three simulated antennas, usually serving as a figure of merit (FOM) for standard FP-LWA antennas. The rather constant value of this FOM (approximately 3.1) indicates a tradeoff between directivity and bandwidth, in analogy to FP-LWAs.

**Supplementary Table 1 | Radiation characteristics of cavity-excited HMS antennas radiating towards $\theta_{out} = 30°$ (*cf.* Supplementary Fig. 1).**

| | $L = 10\lambda\left(d = 1.61\lambda, \lvert z'\rvert = 1.00\lambda, \xi_{out} = \frac{5}{36}\pi\right)$ | | | $L = 14\lambda\left(d = 1.89\lambda, \lvert z'\rvert = 1.33\lambda, \xi_{out} = \frac{2}{36}\pi\right)$ | | |
|---|---|---|---|---|---|---|
| | Full-wave | Semianalytical | Uniform | Full-wave | Semianalytical | Uniform |
| **Main beam** | 30.2° | 30.0° | 30.0° | 30.2° | 30.0° | 30.0° |
| **HPBW** | 6.20° | 5.90° | 5.86° | 4.12° | 4.20° | 4.20° |
| **Directivity [dBi]** | 16.54 | 17.23 | 17.36 | 18.12 | 18.49 | 18.82 |
| **Side lobe #1** | 20.55° | 20.49° | 20.49° | 23.32° | 22.96° | 23.13° |
| **Side lobe #1 level [dB]** | −13.7 | −11.4 | −13.5 | −9.0 | −9.7 | −13.5 |
| **Side lobe #2** | 41.04° | 40.54° | 40.54° | 37.42° | 37.59° | 37.38° |
| **Side lobe #2 level [dB]** | −12.0 | −12.3 | −13.5 | −10.2 | −10.4 | −13.5 |

Results for antennas with aperture lengths of $L = 10\lambda$ and $L = 14\lambda$ are presented, exhibiting, respectively, simulated aperture illumination efficiencies of $\eta_{apt} = 83\%$ and $\eta_{apt} = 85\%$, and HPBWs within $94\%$ and $101\%$ of the optimum corresponding to uniform illumination. Side lobes #1 and #2 refer, respectively, to the first side lobes at angles lower and higher than the main beam angle.

**Supplementary Table 2 | Radiation characteristics of a cavity-excited HMS antenna with reduced side-lobe level (*cf.* Supplementary Fig. 2).**

| | $L=10\lambda\left(d=1.61\lambda,\lvert z'\rvert=1.25\lambda\right)$ | | |
|---|---|---|---|
| | Full-wave | Semianalytical | Uniform |
| **HPBW** | 6.13° | 5.89° | 5.08° |
| **Directivity [dBi]** | 17.04 | 17.62 | 17.98 |
| **First side lobe** | 9.62° | 9.21° | 8.2° |
| **Side lobe level [dB]** | –20.7 | –20.5 | –13.5 |

Results for an antenna with aperture length of $L=10\lambda$ are presented, exhibiting simulated aperture illumination efficiency of $\eta_{\text{apt}}=81\%$ and HPBW within $83\%$ of the optimum corresponding to uniform illumination. The simulated first side-lobe level is $\sim$10dB smaller compared to the $L=10\lambda$ antenna designed for maximal directivity and presented in Fig. 6.

## SUPPLEMENTARY NOTE 1: DESIGN PROCEDURE ASSUMPTIONS

Several assumptions made during the derivation of the HMS design formulas contribute to discrepancies between predicted and actual performance of the presented antennas. First, the predicted fields are derived assuming the HMS is capable of implementing continuous surface impedance boundary conditions, with unbound surface impedance values; nevertheless, the physical implementation requires discretization of the continuous modulation into unit-cells, and the range of achievable surface impedance values is limited (See Supplementary Fig. 4). Second, the HMS is assumed to be passive and lossless, however realistic conductors and dielectrics, used for the implementation of the devices in ANSYS HFSS, include unavoidable losses. Third, to facilitate the plane-wave-like relation between the transmitted fields on the aperture, while still guaranteeing they obey Maxwell's equations, we have used the approximation

(1) $$\left|\mathcal{F}^{-1}\left\{\tfrac{1}{2\beta}T\left(k_t\right)\right\}\right| \approx \left|\mathcal{F}^{-1}\left\{\tfrac{1}{2\beta}T\left(k_t\right)\left[1\pm\Gamma\left(k_t\right)\right]\right\}\right|$$

or, equivalently,

(2) $$\mathcal{E}\left(y\right)=\left|\frac{\mathcal{F}^{-1}\left\{\tfrac{1}{2\beta}T\left(k_t\right)\Gamma\left(k_t\right)\right\}}{\mathcal{F}^{-1}\left\{\tfrac{1}{2\beta}T\left(k_t\right)\right\}}\right| << 1,$$

which is a refinement of the slowly-varying envelope (SVE) approximation utilized in [1]. This approximation is self-consistent with our design scheme, as when the transmitted fields are directive towards $\theta_{\mathrm{out}}$ as desirable , the dominant components of the transmission spectrum $T\left(k_t\right)$ are in the vicinity of $k_t = k\sin\theta_{\mathrm{out}}$, where the reflection coefficient $\Gamma\left(k_t\right)$ completely vanish (the numerator of Supplementary Equation (2) vanishes).

Interestingly, the impacts of these three assumptions can be assessed by reviewing the predicted and simulated power flow across the metasurface. The $z$-directed power profiles below (blue) and above (red) the HMS as predicted by the semaianalytical formalism (open circles and squares, respectively) and as extracted from full-wave simulations (dashed and solid lines, respectively) are presented in Supplementary Fig. 7, for the three antennas reported in the main text. The fact that the general trend and quantitative data of the semianalytical and simulated results compare well (note that the profiles are plotted using a common $\eta\left(I_0/\lambda\right)^2$ unit scale), indicates that the first assumption is valid. The semianalytical predictions made based on a homogenized continuous surface impedance boundary conditions mostly agree with the simulation data recorded $\lambda/10$ below and above the metsurface, where effective medium theory predicts discretization effects to be negligible [1],[2].

Violations of the second assumption, regarding the lossless nature of the HMS, would manifest themselves as differences between the simulated power profile below and above the metasurface, which must originate in dissipation in the unit-cell conductors and dielectrics. On the other hand, violations of the third assumption, related to the SVE

approximation, would manifest themselves as differences in the semianalytically predicted power profile below and above the metasurface, as they correspond to violations of local power conservation [1].

While local deviations from these two assumptions are found to be rather small, they contribute to a non-negligible reduction of the total power flow across the metasurface (integrated over the aperture length). The values denoted in the legends of Supplementary Fig. 7 indicate that according to full-wave simulations about $15\%$ of the power below the HMS is dissipated in the lossy conductors and dielectrics, while the semianalytically predictions reveal about $10\%$ discrepancy between the power below and above the metasurface.

While these relative deviations can be considered small albeit non-negligible, it seems that they actually balance each other. The theoretical derivation assumes and prescribes a lossless HMS, but the minor violations of the SVE approximation contribute to predicted (Maxwellian) fields which must be supported by small losses. On the other hand, the implemented HMS does include realistically unavoidable losses, which turn out to dissipate a comparable amount of power. We hypothesize that this balance allows overcoming the minor deviations from the theoretical assumptions, facilitating the very good agreement between predicted and simulated results reported in this paper.

## SUPPLEMENTARY NOTE 2: EXTENSION TO 3D CONFIGURATIONS AND POLARIZATION CONTROL

The cavity-excited HMS antennas proposed in the manuscript are designed to radiate TE-polarized fields from a 2D configuration. However, the general formalism to design HMSs which convert arbitrary sources to directive radiation [1] can be extended to 3D structures (e.g. with a circular or rectangular apertures) which also allow polarization control. Such an extension was presented in [3] for designing HMS-based devices radiating at broadside, relying on a 3D spectral representation of the fields (using a 2D Fourier transform), local impedance equalization, and local power conservation (similar to [1]). To allow arbitrary source-field polarizations and to provide polarization control of the radiating fields, the formalism indicates that the HMS should consist of anisotropic unit cells, similar to [4].

There are two main points that have to be addressed in order to allow design of 3D cavity-excited HMS antennas as described in the previous paragraph. First, the cavity optimization scheme, intended to enhance the excitation of the high-order lateral mode, has to be adjusted to accommodate circular or rectangular lateral cavities. Similar to the design procedure outlined in the main text, once the (now 3D) source spectrum is assessed, the rest of the design becomes straightforward. Following the same reasoning presented in the main text, it is expected that if high-order lateral modes (note that now there are two lateral dimensions) are predominantly excited, high aperture illumination efficiencies can be achieved from circular or rectangular apertures as well. Second, a lookup table for anisotropic Huygens' meta-atoms has to be constructed for operation in the design frequency. This may be somewhat simplified by considering the fact that the meta-atoms can be synthesized by rotating anisotropic unit cells whose primary axes coincide with the $x$ and $y$ axes of Fig. 1 [3], [4]. It should be noted that such anisotropic unit cells have already been demonstrated at other microwave frequencies [5], serving as a good starting point for implementing the required surface impedance tensors. In conclusion, we believe that 3D cavity-excited HMS antennas can be designed by combining the methodology presented herein with the 3D formalism of [3], and realized via anisotropic physical design based on previous work [5].

Considering that the manuscript focuses on developing and elucidating the physical concept underlying cavity-excited HMS antennas, detailed design and implementation of 3D antennas are left for future work. Dual-polarized operation, which may also be of interest for advanced applications, will be more suitably investigated in the framework of such a 3D design procedure, where polarization aspects have to be explicitly addressed.

# SUPPLEMENTARY METHODS

## DESIGN OF CAVITY-EXCITED HMS ANTENNAS RADIATING AT OBLIQUE ANGLE

To design cavity-excited HMS antennas for radiating at oblique angle we follow the same procedure outlined in the main text for broadside radiators, with the given $\theta_{\rm out} \neq 0$ inserted into the formulas; note that now $Z_{\rm out} = 1/Y_{\rm out} = \eta / \cos\theta_{\rm out} \neq \eta$. The same argumentations for optimizing the cavity excitation hold (Figs. 2 and 3), where the HMS transmission spectra plotted in Fig. 2e merely shifted by $k_t = k \sin\theta_{\rm out}$. This is essentially identical to the design of antenna arrays radiating at an angle $\theta_{\rm out}$, where the magnitude of the array element currents are usually determined independently of $\theta_{\rm out}$, and the direction of the main beam is manifested via phase-shifts imposed between adjacent elements [6]. Therefore, the optimal configuration still follows equation (7), i.e. we use the same cavity excitation regardless of $\theta_{\rm out}$. Once the required electric surface impedance and magnetic surface susceptance are specified, the corresponding spider unit-cell dimensions are retrieved from a lookup table constructed as described in the Methods, with $Z_{\rm out} = 1/Y_{\rm out} = \eta / \cos\theta_{\rm out}$.

We use this outlined procedure to design cavity-excited HMS antennas radiating towards $\theta_{\rm out} = 30°$; the corresponding lookup table is graphically presented in Supplementary Fig. 3. The design specifications and radiation characteristics are presented in Supplementary Table 1 and Supplementary Fig. 1 for devices with aperture lengths of $L = 10\lambda$ and $L = 14\lambda$, comparing the results of full-wave simulations, semianalytical predictions, and uniformly-excited apertures (note that for the latter, HPBW and directivity are factored by $\cos\theta_{\rm out}$ with respect to their values for broadside radiation). In consistency with the aforementioned decoupling between the excitation and main-beam angle, the optimal cavity configuration for maximal directivity is identical to the one derived for broadside radiation (compare Table I and Supplementary Table 1). Indeed, the Supplementary Fig. 1e-h show that the field profile inside the cavity is practically independent of $\theta_{\rm out}$ (*cf.* Fig. 6).

To match better the range of required surface impedance values (Supplementary Fig. 1) to the one achievable by our spider unit-cells (Supplementary Fig. 4), we introduce a constant phase-shift $\xi_{\rm out}$ to the aperture fields [1]. This adds a constant $\xi_{\rm out}/2$ to the argument of the cotangent surface impedance modulation, which varies the distribution of required surface impedance values, but does not affect the radiation pattern.

Simulation methodology follows Methods, with the difference that the PMC in the $xz$ plane cannot be used, as the HMS is not symmetric in this case (Supplementary Fig. 1a-b). This doubles the volume of the simulation domain, resulting in insufficient convergence of the computations for $L = 25\lambda$ devices. Hence, only results for $L = 10\lambda$ and $L = 14\lambda$ are presented in Supplementary Table 1 and Supplementary Fig. 1.

## DESIGN OF CAVITY-EXCITED HMS ANTENNAS WITH REDUCED SIDE-LOBE LEVEL

As denoted in the main text, for an optimized cavity thickness $d$, i.e. one that maximizes coupling to the highest-order mode, one may utilize the source position $z'$ as an additional degree of freedom to optimize the antenna radiation characteristics to achieve desirable performance. In the main text, we utilized this degree of freedom to suppress the coupling to the lowest-order mode, in order to facilitate the highest possible directivity. This, in consistency with array theory [6], comes at the expense of side-lobe level.

To demonstrate the possibility to utilize the efficient semianalytical formulation to devise a cavity excitation which reduces the side-lobe level, we sweep the source position $z'$ for the optimal $d = 1.61\lambda$ corresponding to an aperture length of $L = 10\lambda$. As we only require examination of the radiation pattern properties for each value of $z'$, it is sufficient to compute the radiated fields, which can be achieved analytically by asymptotic evaluation of equation (2) in conjunction with equations (4) and (6) (See, e.g. Appendix C of [1]).

The results of the parametric sweep are presented in Supplementary Fig. 3, with indication of the maximal directivity configuration ($|z'| = 1\lambda$) and a configuration with reduced side-lobe level ($|z'| = 1.25\lambda$). The design specifications and radiation characteristics of the cavity-excited HMS antenna corresponding to the latter are presented in Supplementary Fig. 2a-d and Supplementary Table 2. As expected from array theory, the reduction of side-lobe level (by ~10dB according to full-wave simulations) results in a broadening of the main beam and a reduction of the overall aperture illumination efficiency (although still quite high value of $\eta_{\mathrm{apt}} = 81\%$ is achieved with full-wave simulations). This demonstrates the versatility of our approach, with which a range of radiation pattern properties can be achieved.

## EXPERIMENTAL EVALUATION OF GAIN, DIRECTIVITY, RADIATION EFFICIENCY AND APERTURE ILLUMINATION EFFICIENCY

The 3D realized gain was evaluated from far-field measurements following the gain-comparison method. In the framework of this methodology, two standard-gain horns with known gain characteristics are aligned and measured to obtain frequency-dependent calibration factors for successive measurements. Subsequently, the power received by the antenna under test (AUT) when positioned in front of the transmitting standard-gain horns would corresponds, after using these calibration factors, to the AUT realized gain.

Since the gain-comparison method is quite sensitive to the mutual alignment of the transmitting and receiving antennas, both in the calibration and testing stages, we include an error margin of $\pm 1\mathrm{dB}$ in the value of the realized gain, estimated based on past experience. Therefore, to evaluate the realized gain of the AUT we have performed two separate calibration and testing measurements, at two different times, and used the average realized gain as our best estimate, accompanied with the aforementioned error margin.

The 3D gain $G_{3\mathrm{D}}$ is calculated from the realized gain by dividing it by $(1-R)$, with $R$ being the (power) reflection coefficient of the AUT (inversely proportional to the return loss) [7], obtained via vector network analyzer (VNA) measurements. In order to evaluate the radiation efficiency $\eta_{\mathrm{rad}} = G_{3\mathrm{D}} / D_{3\mathrm{D}}$, the 3D directivity $D_{3\mathrm{D}}$ should be evaluated.

To evaluate $D_{3\mathrm{D}}$ we have measured the radiation patterns of the AUT in three different cuts: across the $yz$, $xz$, and $xy$ planes, corresponding to the $\Phi = \pm\pi/2$, $\Phi = 0,\pi$, and $\Theta = \pi/2$ planes, respectively, where $\{(\Theta,\Phi) \,|\, \Theta \in [0,\pi], \Phi \in [-\pi,\pi]\}$ being the standard spherical coordinate system (i.e. $\Theta$ is measured with respect to the $z$ axis and $\Phi$ is measured with respect to the $x$ axis, as these axes are defined in Fig. 1). To reconstruct the 3D radiation pattern we have used the $yz$ and $xz$ radiation patterns as reference data points, and interpolated the radiation pattern assuming the radiation from a cavity-excited HMS antenna would possess symmetry properties with respect to the $y$ and $z$ axes.

To be more specific, the normalized radiation pattern $\bar{S}_r(\Theta,\Phi)$ measured at the $yz$ plane provides the data points $\{\bar{S}_r(\Theta,\pi/2), \bar{S}_r(\Theta,-\pi/2) \,|\, \Theta \in [0,\pi]\}$, and the $xz$ plane measurements provide $\{\bar{S}_r(\Theta,0), \bar{S}_r(\Theta,\pi) \,|\, \Theta \in [0,\pi]\}$. The aforementioned radiation symmetry implies that the radiation at $(\Theta,\Phi)$ would be proportional to the normalized radiation at $(\Theta_x,0)$ or $(\Theta_x,\pi)$ and the normalized radiation at $(\Theta_y,\pm\pi/2)$, where $\Theta_x,\Theta_y \in [0,\pi/2]$ satisfy

$$\begin{aligned} \sin\Theta_x &= \sin\Theta\left|\cos\Phi\right| = \left|k_x\right|/k \\ \sin\Theta_y &= \sin\Theta\left|\sin\Phi\right| = \left|k_y\right|/k \end{aligned}, \quad (3)$$

where $k_x$ and $k_y$ are the projections of the wave vector of the far-field ray reaching $(\Theta,\Phi)$ on the $x$ and $y$ axes, respectively. The interpolated (normalized) radiation pattern thus reads

$$(4)\qquad \bar{S}_r(\Theta,\Phi)=\begin{cases}\bar{S}_r(\Theta_x,0)\bar{S}_r(\Theta_y,\pi/2) & \Theta\in[0,\pi/2],\Phi\in[0,\pi/2]\\ \bar{S}_r(\Theta_x,0)\bar{S}_r(\Theta_y,-\pi/2) & \Theta\in[0,\pi/2],\Phi\in[-\pi/2,0]\\ \bar{S}_r(\Theta_x,\pi)\bar{S}_r(\Theta_y,\pi/2) & \Theta\in[0,\pi/2],\Phi\in[\pi/2,\pi]\\ \bar{S}_r(\Theta_x,\pi)\bar{S}_r(\Theta_y,-\pi/2) & \Theta\in[0,\pi/2],\Phi\in[-\pi,-\pi/2]\\ \bar{S}_r(\pi-\Theta_x,0)\bar{S}_r(\Theta_y,\pi/2) & \Theta\in[\pi/2,\pi],\Phi\in[0,\pi/2]\\ \bar{S}_r(\pi-\Theta_x,0)\bar{S}_r(\Theta_y,-\pi/2) & \Theta\in[\pi/2,\pi],\Phi\in[-\pi/2,0]\\ \bar{S}_r(\pi-\Theta_x,\pi)\bar{S}_r(\Theta_y,\pi/2) & \Theta\in[\pi/2,\pi],\Phi\in[\pi/2,\pi]\\ \bar{S}_r(\pi-\Theta_x,\pi)\bar{S}_r(\Theta_y,-\pi/2) & \Theta\in[\pi/2,\pi],\Phi\in[-\pi,-\pi/2]\end{cases}$$

where the normalization is always with respect to the radiation towards the positive $z$ direction $(0,\Phi)$, which is common to the two radiation patterns.

The interpolated 3D radiation pattern for the measurement working frequency $f=20.04\text{GHz}$ is presented in Supplementary Fig. 6a. Supplementary Figures 6b-d compare the measured patterns in the $yz$, $xz$, and $xy$ plane cuts to the patterns evaluated using the interpolation scheme. We note that even though *we did not use* the $xy$ cut data points for the interpolation, nor the points with $|\Theta|>\pi/2$ in the $yz$ cut, Supplementary Figs. 6b,c show that the interpolated values agree well with the measured ones. This provides further support to the validity of our symmetry assumptions made to establish the interpolation procedure.

With the interpolated 3D radiation pattern in hand, the 3D directivity could be readily calculated using

$$(5)\qquad D_{\text{3D}}=\frac{4\pi\max\left\{\bar{S}_r(\Theta,\Phi)\right\}}{\int_0^{\pi}\sin\Theta d\Theta\int_{-\pi}^{\pi}d\Phi\bar{S}_r(\Theta,\Phi)}$$

Finally, the radiation efficiency can be evaluated via $\eta_{\text{rad}}=G_{\text{3D}}/D_{\text{3D}}$, with the appropriate error intervals included in the value of $G_{\text{3D}}$ as discussed before. The radiation efficiency obtained following this procedure is presented in Supplementary Fig. 5b. It should be noted that the error bars presented therein only correspond to errors related to the gain-comparison method, and that in general, radiation efficiency values calculated away from the design frequency, where the reflection coefficient is high and the gain and directivities are low, are expected to be less accurate [7].

As a final comment, we note that the measured aperture illumination efficiency values $\eta_{\mathrm{apt}} = D/(2\pi L/\lambda)$ presented in the main text refer to the 2D directivity calculated over the $yz$ cut radiation in the positive $z$ direction, i.e.

(6) $$D = \frac{2\pi \max\left\{\bar{S}_r\left(\Theta, \pm\pi/2\right)\right\}}{\int_0^{\pi/2} \bar{S}_r\left(\Theta, \pi/2\right) d\Theta + \int_0^{\pi/2} \bar{S}_r\left(\Theta, -\pi/2\right) d\Theta}$$

to allow fair comparison with the 2D theory and full-wave simulations, where backwards radiation is not allowed. For completeness, however, we present in Supplementary Fig. 5a also the aperture illumination efficiency corresponding to $D_{2\pi}$, calculated from the entire radiation pattern, i.e. following

(7) $$D_{2\pi} = \frac{2\pi \max\left\{\bar{S}_r\left(\Theta, \pm\pi/2\right)\right\}}{\int_0^{\pi} \bar{S}_r\left(\Theta, \pi/2\right) d\Theta + \int_0^{\pi} \bar{S}_r\left(\Theta, -\pi/2\right) d\Theta}$$

As can be observed, the measured backwards radiation is minor, and the inclusion of both the positive and negative $z$ directions does not significantly affect the evaluated aperture illuminated efficiency. For instance, for $f = 20.04\mathrm{GHz}$, the aperture illumination efficiency calculated from $D$ is 75%, whereas the aperture illumination efficiency calculated from $D_{2\pi}$ is 73%.

## SUPPLEMENTARY REFERENCES


[1] Epstein, A. & Eleftheriades, G. V. Passive lossless Huygens metasurfaces for conversion of arbitrary source field to directive radiation", *IEEE Trans. Antennas Propag.*, **62**, 5680-5695 (2014).

[2] Tretyakov, S. A., *Analytical Modeling in Applied Electromagnetics* (Artech House, 2003).

[3] Epstein, A. & Eleftheriades, G. V. Coupling localized sources to controlled polarized broadside radiation using Huygens metasurfaces. In *Proc. IEEE Int. Symp. Antennas and Propagation (APSURSI)*, (Vancouver, BC, 2015).

[4] Selvanayagam, M. & Eleftheriades, G. V. Polarization control using tensor Huygens surfaces. *IEEE Trans. Antennas Propag*. **62**, 6155-6168 (2014).

[5] Pfeiffer, C. & Grbic, A. Controlling vector Bessel beams with metasurfaces*. Phys. Rev. Appl.* **2**, 044012 (2014).

[6] Tsoilos , G. V. & Christodoulou, C. G. ,*Modern antenna handbook* (Wiley, Hoboken, NJ, 2008) Chap. 11.

[7] Pfeiffer, C. & Grbic, A. Planar lens antennas of subwavelength thickness: Collimating leaky-waves with metasurfaces*. IEEE Trans. Antennas Propag.* **63**, 3248-3253 (2015).
